# Supplementary material for: Local and global reward learning in the lateral frontal cortex show differential development during human adolescence
Source: PLoS Biol. 2023 Mar 2;21(3):e3002010. doi: 10.1371/journal.pbio.3002010 (PMC10013901; doi:10.1371/journal.pbio.3002010)
Supplement: S1 Text — Supplementary Material: Fig A. Influence of choice history on switch/stay decisions does not change across adolescence. As a control analysis, we considered developmental changes in a reward-unrelated learning mechanism that was also included in our credit assignment GLM. We examined C-history, the tendency to repeat choices irrespective of reward [1]. In general, participants were more likely to repeat the most recent choice, irrespective of reward (one-sample t test; Ct-1: t352 = 3.09, p = 0.002). (A) However, Ct-1 did not differ between adolescents and adults (independent samples t test; t351 = 1.16, p = 0.245). (B) Analogously, there was no correlation between age and Ct-1 (R = 0.07, p = 0.191). (“x”s indicate individual participants; plots show mean −/+ SEM; solid line in the right plots indicates best fitting linear trend. Dashed lines represent 95th% confidence interval). Data for B and C are available in S1 Data (Fig A tab). Fig B. No developmental changes in decision computations. Complementing our analyses of global and local reward learning, we also considered developmental changes in decision-related computations. (A) We first fitted a simple reinforcement learning model to our data. This model was fitted individually to each session’s data using standard nonlinear minimization procedures and a Boltzmann action selection rule. In line with the CxRt effects reported above, learning rates for young adults were significantly higher than for adolescents (independent samples t test, t386 = −3.83, p < 0.001). (B) This result was confirmed as a significant correlation with age (Pearson correlation, R = 0.19, p < 0.001). (C) Notably, the age groups did not differ in their general levels of decision-making noise, as the RL models’ (log-normalised, to account for outliers) inverse temperature parameter did not differ with age (independent samples t test, t379 = −1.83, p = 0.068; Pearson correlation, R = 0.02, p = 0.720). Note the effect remained nonsignificant whe [file pbio.3002010.s001.docx]

**Local and global reward learning in the lateral frontal cortex show differential development during human adolescence**

Marco K. Wittmann^1,2,3^^,4 ¶^, Maximilian Scheuplein^1,5 ¶^, Sophie G. Gibbons^1,6^, MaryAnn P. Noonan^1,7*^

^1^ Department of Experimental Psychology, University of Oxford, Radcliffe Observatory,,, Oxford , UK.
^2^ Wellcome Centre for Integrative Neuroimaging, University of Oxford, John Radcliffe Hospital, , Headington, Oxford , UK
^3^ Department of Experimental Psychology, University College London, , London , UK
^4^ Max Planck UCL Centre for Computational Psychiatry and Ageing Research, University College London, UK

^5^ Institute of Education and Child Studies, Leiden University, Leiden, The Netherlands.

^6^ MRC Cognition and Brain Sciences Unit, University of Cambridge, Cambridge , UK.

^7^ Department of Psychology, University of York, York, UK.

^¶^ authors contributed equally to this work.

^*^[maryann.noonan@psy.ox.ac.uk](mailto:maryann.noonan@psy.ox.ac.uk)

**Supporting Information: S1 Text**

**Supplementary Material**


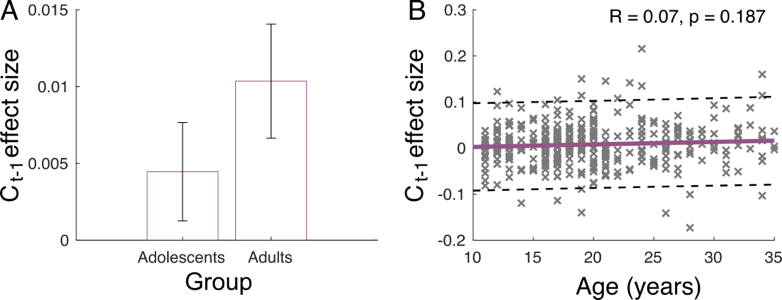


**Fig A. Influence of choice history on switch/stay decisions does not change across adolescence.** As a control analysis, we considered developmental changes in a reward-unrelated learning mechanism that was also included in our credit assignment GLM. We examined C-history, the tendency to repeat choices irrespective of reward [1]. In general, participants were more likely to repeat the most recent choice, irrespective of reward (one-sample t-test; C_t-1_: t_352_=3.09, p=0.002). **(A)** However, C_t-1_ did not differ between adolescents and adults (independent samples t-test; t_351_=1.16, p=0.245). **(B)** Analogously, there was no correlation between age and C_t-1_ (R=0.07, p=0.191). (“x”s indicate individual participants; plots show mean -/+SEM; solid line in the right plots indicates best fitting linear trend. Dashed lines represent 95^th^% confidence interval). Data for B and C are available in S1 Data (Fig A tab).


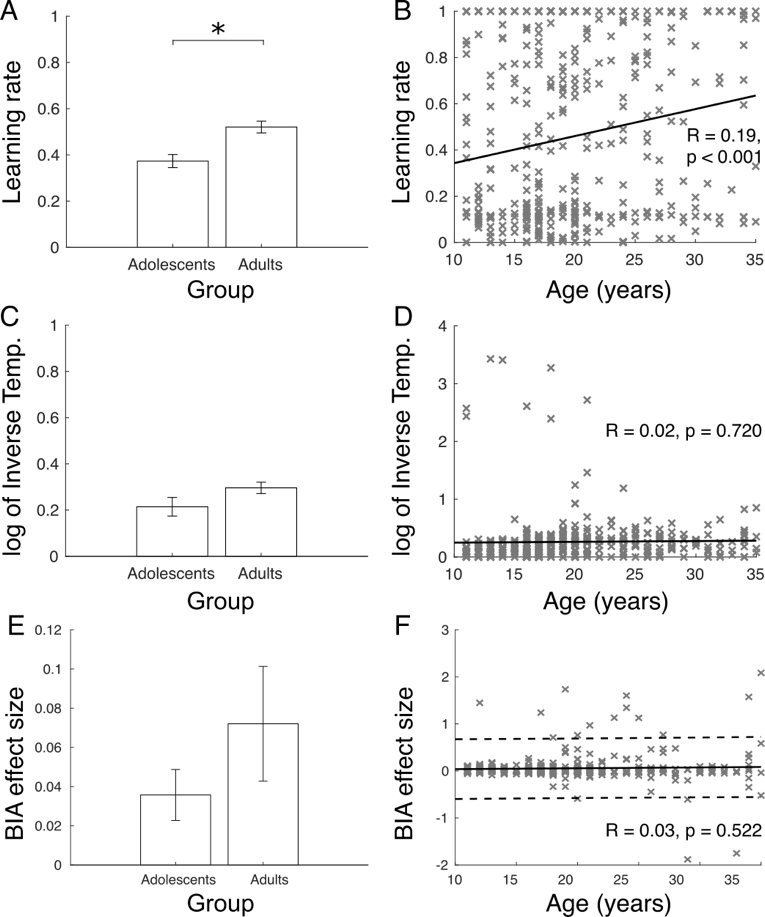


**Fig B. No developmental changes in decision computations.** Complementing our analyses of global and local reward learning, we also considered developmental changes in decision-related computations. **(A)** We first fitted a simple reinforcement learning model to our data. This model was fitted individually to each session’s data using standard nonlinear minimization procedures and a Boltzmann action selection rule. In line with the CxR_t_ effects reported above, learning rates for young adults were significantly higher than for adolescents (independent samples t-test, t_386_=-3.83, p<0.001). **(B)** This result was confirmed as a significant correlation with age (Pearson correlation, R=0.19, p<0.001). **(C)** Notably, the age groups did not differ in their general levels of decision-making noise, as the RL models’ (log-normalized, to account for outliers) inverse temperature parameter did not differ with age (independent samples t-test, t_379_=-1.83, p=0.068; Pearson correlation, R=0.02, p=0.720). Note the effect remained non-significant when the inverse temperature was not log normalized. This suggests that changes in learning rates cannot be reduced to changes in decision noise. It furthermore also strongly indicates that our previous results about GRS-related maturation are not driven by differences in decision noise between the age groups. **(D)** Examining the developmental trajectory of this parameter over time also failed to reveal a significant change. **(E,F)** Finally, following our analysis approach established in human medial frontal lesion patients [2], we used a combination of multinomial logistic regression analysis and reinforcement learning modelling (see above) to examine the influence of a value-based decision bias. We considered each three-choice decision as two binary comparisons and rearranged them such that we can extract the biasing effect of the value of a distractor option on choice. Using these expected values generated for each option on each trial we examined whether the interactive impact of the decision-irrelevant option’s value (V_D_) on the choice between the two relevant Options (V_X_ and V_Y_). We applied a two-step multinomial logistic regression analysis, that has been described in full, alongside the complete set of equations, in Noonan et al. (2017). We chose this specific GLM to make the findings directly comparable to our previous human lesion study. In short, this approach reframes the 3-choice decision as two binary value comparisons between pairs of options. The GLM aims to predict the proportion of choices among the three options from their expected values, with one option assigned in each decision frame as the reference category. For example, Option X and Y are the options being compared; with Option Y as the reference, Option X as the comparator and Option D denoting the irrelevant option. Each option’s values (V_X_, V_Y_, V_D_) were initially derived from a reinforcement learning model described above. The present study examines distractor effects on choice as a function of potential regional differences in the speed of brain maturation during adolescence. Previous lesion studies have characterised this as a negative influence [2,3] and so we selected a model that allowed us to focus on that specific factor. The key step in the model, for the purposes of the present study is the isolation of the contextual decision-making factor (V_X_ - V_Y_)V_D_ from the final step of the GLM outlined in equation 1 (equation 7 in [2]). Intuitively, this term reflects the modulation of the decision variable (the value difference between the options) by the distractor.

$\ln\left( \frac{P\left( X \right)}{P\left( Y \right)} \right)=\beta_{0}+\frac{{(\beta}_{1}-\beta_{2})}{2}{(V}_{X}-V_{Y})+\frac{{(\beta}_{1}+\beta_{2})}{2}{(V}_{X}+V_{Y})+\beta_{3}V_{D}+\beta_{4}V_{X}V_{Y}+\frac{{(\beta}_{5}-\beta_{6})}{2}{(V}_{X}-V_{Y})V_{D}+\frac{{(\beta}_{5}+\beta_{6})}{2}{(V}_{X}+V_{Y})V_{D}$ (1)

This factor allows us to examine how the expected value of the irrelevant option V_D_ affects the comparison between X and Y (i.e. $\frac{{(\beta}_{5}-\beta_{6})}{2}{(V}_{X}-V_{Y})V_{D}$), after controlling for the effects of the difference between the two options (V_X_-V_Y_), their total value (V_X_+V_Y_) and their interaction (V_X_×V_Y_), as well as the independent value of the distractor (V_D_) and the interaction between the distractors value and the relevant options’ combined value ((V_X_+V_Y_)V_D_). In other words, the (V_X_ - V_Y_)V_D_ beta weight reflects the degree to which the effect of value difference between X and Y on choices between these two options was modulated by the irrelevant distractor value (V_D_). For brevity, we refer to our variable of interest, the (V_X_ - V_Y_)V_D_, as *bias by irrelevant alternative* (BIA). In addition to the standard exclusion criteria, the regression model described below failed to fit a total of 32 participants and were excluded from this analysis. Subsequently, the factor isolated from the GLM was subjected to an outlier rejection procedure (15 participants), and the beta weights were absolute log transformed. Beta weights were then submitted to a second-level age-comparison analyses. The current choice data showed that BIA did not differ with age (independent samples t-test, t_339_=1.06, p=0.291; Pearson correlation, R=0.03, p=0.522). Therefore, in contrast to the local and global reward learning mechanisms linked to lateral prefrontal cortex, the influence of the value of third option on the binary choice may already reflect a matured functional state by the age of our sample. (“x”s indicate individual participants; plots show mean -/+SEM; solid line in the right plots indicates best fitting linear trend. Dashed lines represent 95^th^% confidence interval. * p<0.05). Data for A-F are available in S1 Data (Fig B tab).

**
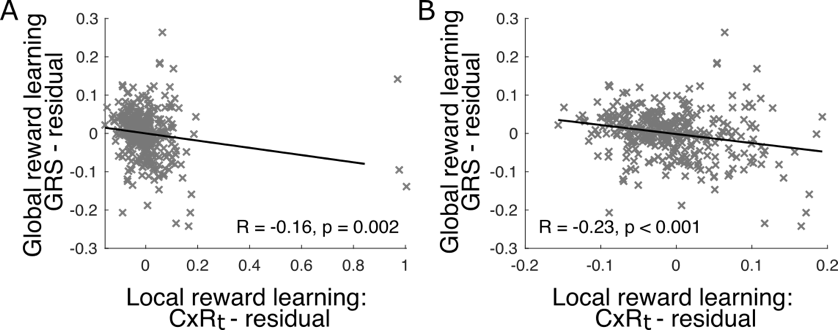
**

**Fig C. Local and global reward learning correlated across participants.** We investigated the relationships between local reward assignments and negative GRS effects. Despite the theoretical accounts arguing that a negative GRS effect might aid value learning, it could be argued that GRS effects per se are suboptimal in the context of probabilistic learning tasks. To address this, we examined the relationship of GRS with a marker of local contingent value assignment, the CxR_t_ effect, as the latter reflects a signature of successful learning in this task. Controlling for participant age and their GLM constant, we examined the relationship between GRS and RxC_t_ using a partial correlation. **(A)** Our findings revealed a strong negative correlation between contingent reward assignment and the global reward effect (Pearson corelation, R=-0.16, p=0.002). This suggests that individuals who are more influenced by local reward assignment mechanisms are also more likely to rely on a negative reward contextualisation. This pattern of behaviour further supports the idea that negative GRS effects are adaptive and may co-mature with contingent credit assignment mechanisms during adolescence. Visual inspection might suggest that the correlation is potentially driven by three outliers with high contingent learning scores. **(B)** However, removal of these data points confirmed that this was not the case; instead, the correlation became even more significant (R=-0.23, p<0.001). (“x”s indicate individual participants, solid line indicates linear fit). Data for A and B are available in S1 Data (Fig C tab).

*
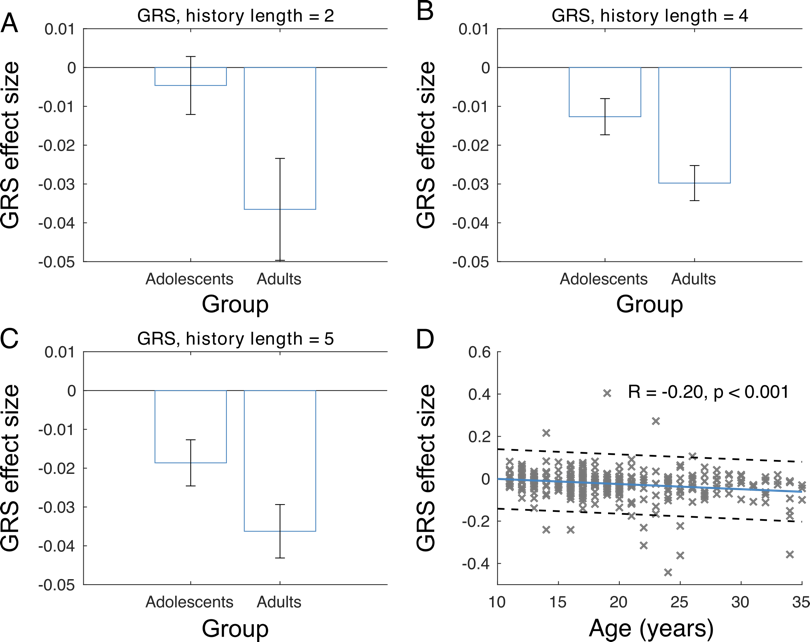
*

**Fig D. The effect of GRS on choice is stable across a broad window of reward history length and does not depend on arbitrary statistical choices**. In our main GLM, the GRS is calculated as the arithmetic mean of rewards occurring during the last three trials (see Methods). This history length was chosen a priori based on previous work and based on the number of trials in the experimental schedule. To show that the GRS effects are stable, we repeated our main GLM and varied the length of this reward history. We varied it between including only the last two trials **(A)**, the last four **(B)** and the last five **(C)**. The respective panels show the GRS effect from these three GLMs. In accordance with varying reward history length, we adjusted the timescale of the other relevant learning mechanisms (CxR-history and C-history) in the GLM. This ensured that the GRS, CxR-history, and C-history were all calculated over the same set of past trials. Consequently, in the analysis, variance associated with one learning mechanism was unlikely to be misattributed to another learning mechanism as they cover the same duration of the trial history. For example, when extending the history length of the GRS to five trials, we also extended the history length of CxR-history and C-history by two trials. We then aggregated these alternative regression models and showed that our effects of interest remained significant. Aggregating the results across the 3 alternative choice history lengths, we compared the beta weights against zero for adolescents and adults separately in two one-sample t-tests and found negative GRS effects both in adolescents (t_153_ = -2.79, p = 0.006) and adults (t_176_ = -5.27, p < 0.001). Importantly, as in our main analysis, adults have a more negative GRS effect than adolescents (F_1,331_ = 8.14, p = 0.005; main effect of age group in 2 [age group: adolescents, adults] x 3 [reward history length: 2,4 or 5] repeated measures ANOVA). Main effects of history length or the interaction between history length and age group were not significant (F_2,666_ = 0.578, p = 0.480, Interaction F_2,666_ = 0.711, p = 0.426). **(D)** Finally, again, as in our main analysis, this developmental trajectory also manifests in a negative correlation between age and GRS effect (r = -0.19, p < 0.001). For this correlation, we averaged the GRS beta weights, within each subject, across the three GLMS with history length 2, 4 and 5. The average GRS beta weights were then plotted against age. Critically, these analyses all used a history length that is different from the one in the main GLM and demonstrate that our results did not depend on arbitrary modelling choices. (“x”s indicate individual participants; plots show mean -/+ SEM; solid line in the right plots indicates best fitting linear trend. Dashed lines represent 95% confidence interval). Data for A-D are available in S1 Data (Fig D tab).


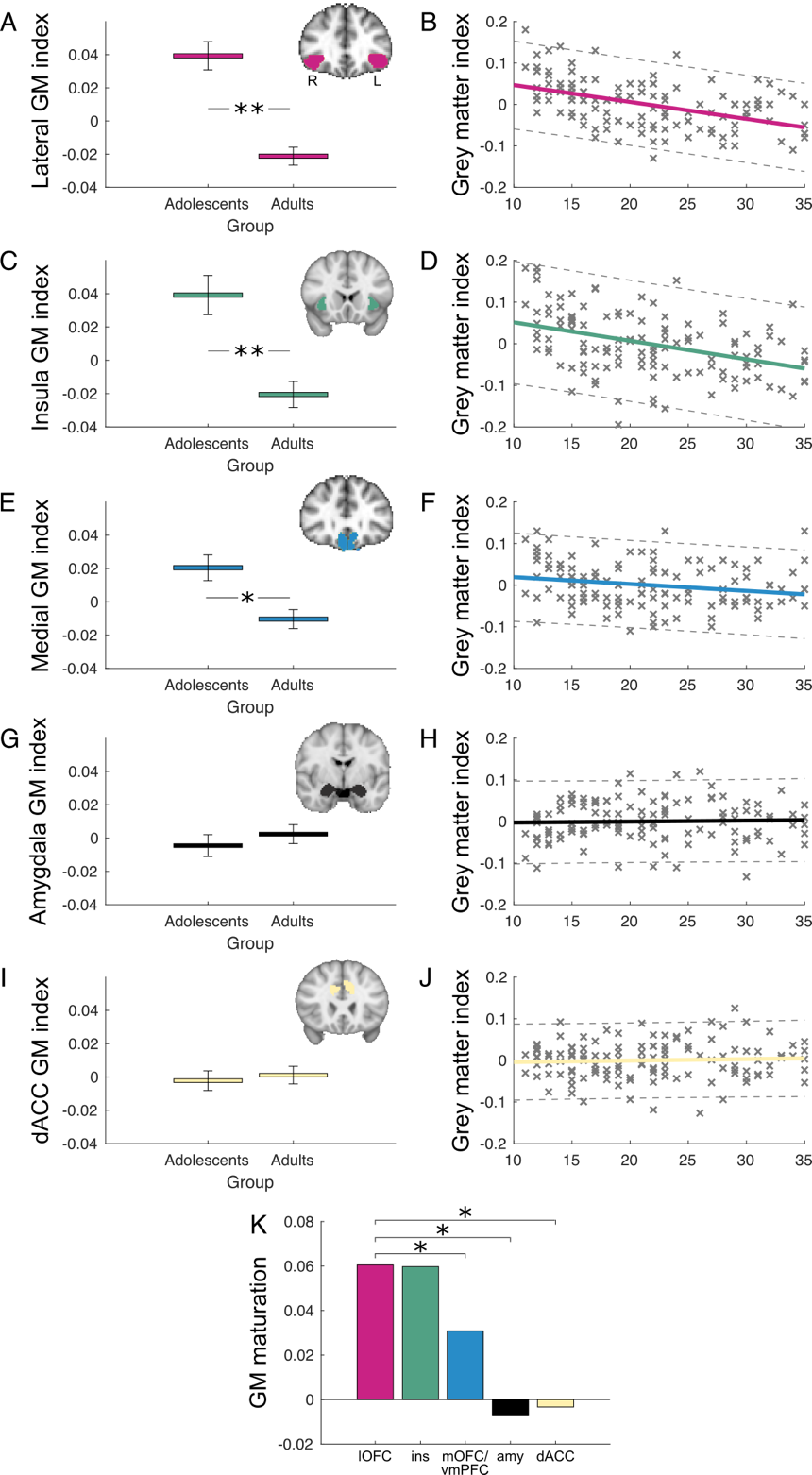


**Fig E. Delayed grey matter maturation in lateral orbitofrontal and anterior insula cortex relative to other networked learning and decision-making neural nodes.** Study 1 showed significant changes in local and global reward learning across adolescent development and into early adulthood. Here we investigated the potential underlying neural changes by examining grey matter maturation in prefrontal cortex during the same time window as our behavioural sample, 11-35 years (see Supplementary Methods). We considered regions of interest (ROI) that are related to reward processing. Local and global components of reward learning have both been previously linked to lateral orbitofrontal (lOFC) and anterior insula cortex (Ins) respectively [1,3–5]. Medial orbitofrontal/ventromedial prefrontal cortex (mOFC/vmPFC) is causally linked to value comparison mechanisms [3,6,7], while the dorsal anterior cingulate (dACC) is associated with learning from feedback with BOLD activity in this region correlated with adapting learning rate [8]. Finally amygdala (amy) grey matter density increases with experience in reversal learning-like tasks such as ours [9], lesions to the amygdala affect reversal learning [10] and amygdala signals deviations from precise local reward learning [11,12]. Guided by past NHP work we analyzed structural brain data from an independent data set of 125 individuals from the Human Connectome Project data (HCP developmental and young adult data [13,14]), evenly spread out across our investigated age range. We conducted this study in parallel to study 1. Estimates of individual participants grey matter thickness were extracted from anatomical masks of lateral orbitofrontal cortex and medial orbitofrontal/ventromedial prefrontal cortex [15], dorsal anterior cingulate cortex, amygdala and anterior insula. Developmental trajectories of all five regions were compared using ANCOVA analysis and showed significant differential GM patterns across age (F_4,492_ = 12.35, p<0.001). Follow-up sub analyses compared lateral orbitofrontal cortex separately with the other four areas. Adults and adolescents were also compared directly in independent samples t-tests and Pearson correlational analyses. **(A,B)** Supporting our hypothesis we showed that grey matter in lateral orbitofrontal cortex was significantly lower in young adults compared to adolescence (independent samples t-test, t_123_=6.23, p<0.001) and correlated negatively with age (Pearson correlation, R=-0.47, p<0.001; Fig.4B), with link functions suggesting this relationship was best fit with a quadratic function (Table A in S1 Text). **(C,D)** The GM trajectory of the anterior insula, a region in which BOLD activity correlates with the GRS in macaques [1,9] also showed a significant relationship with age (independent samples t-test: t_123_ = -4.34, p < 0.001, Pearson correlation, R = -0.39, p < 0.001). Follow-up tests suggest this relationship was best characterised by a quadratic function (Table A in S1 Text). Direct comparison between the GM trajectories of lateral orbitofrontal cortex and the anterior insula revealed no significant differences between the GM trajectory of the two regions (F_1,123_ = 0.16, p = 0.694). **(E,F)** The medial orbitofrontal/ventromedial prefrontal cortex also showed continued maturation across the age-range sampled (independent samples t-test, t_123_=3.19, p=0.002; Pearson correlation, R=-0.21, p=0.018; Fig.4C,D) with model fits again characterising this relationship as quadratic (Table A in S1 Text). However, as the ANCOVA results revealed differential developmental trajectories of GM between lateral and medial orbitofrontal cortex, indexed by a significant age x subregion interaction (F_1,123_=9.896, p=0.002) which suggested medial maturation was significantly less pronounced than lateral regions. **(G,H)** By contrast there was no relationship between age and grey matter in the amygdala, a subcortical region heavily connected with lateral orbitofrontal cortex and intrinsically linked to complementary components of local reward learning [10] (independent samples t-test, t_123_ = -0.79, p = 0.43; Pearson correlation, R = 0.03, p = 0.716; Note this did not improve by using a quadratic instead of a linear link function, see Table A in S1 Text, which replicates past developmental GM studies [16,17]. Direct comparison between the GM trajectories of lateral orbitofrontal cortex and the amygdala showed, as expected, that developmental GM trajectory was significantly more pronounced in lateral prefrontal cortex compared to the amygdala (significant age x region interaction F_1,123_ = 23.37, p <0.001). **(I,J)** Finally, we examined GM trajectory of the anterior cingulate cortex (focusing on the RCZa or more commonly referred to as dorsal ACC). GM in this region did not vary as a function of age (independent samples t-test: t_123_ = -0.75, p = 0.456, Pearson correlation, R = 0.05, p = 0.549). Direct comparison between the GM trajectories of lateral orbitofrontal cortex and the dACC showed, as expected, that developmental GM trajectory was significantly more pronounced in lateral orbitofrontal cortex (significant age x region interaction (F_1,123_ = 24.80, p <0.001). **(K)** Illustration of the between-subjects interaction of the GM maturation (calculated as mean adolescents minus mean adults) between lateral orbitofrontal cortex, medial orbitofrontal/ventromedial prefrontal cortex, amygdala, dorsal anterior cingulate cortex and anterior insula. This highlights the significantly stronger maturation of grey matter in lateral orbitofrontal cortex and anterior insula compared to the other networked brain regions. This pattern suggests the lateral orbitofrontal and anterior insula cortex undergo the most extensive changes during adolescence, findings in line with a general pattern of maturation across adolescence [18,19]. This suggests that cognitive functions supported by these regions may also undergo more pronounced changes during development compared to those supported by the other areas in the learning and decision-making network. (“x”s indicate individual participants; plots show mean -/+SEM; solid line in the right plots indicate a linear fit. Dashed lines represented 95^th^% confidence intervals. * p<0.05, ** p<0.001). Data for A-K are available in S1 Data (Fig E tab).

**
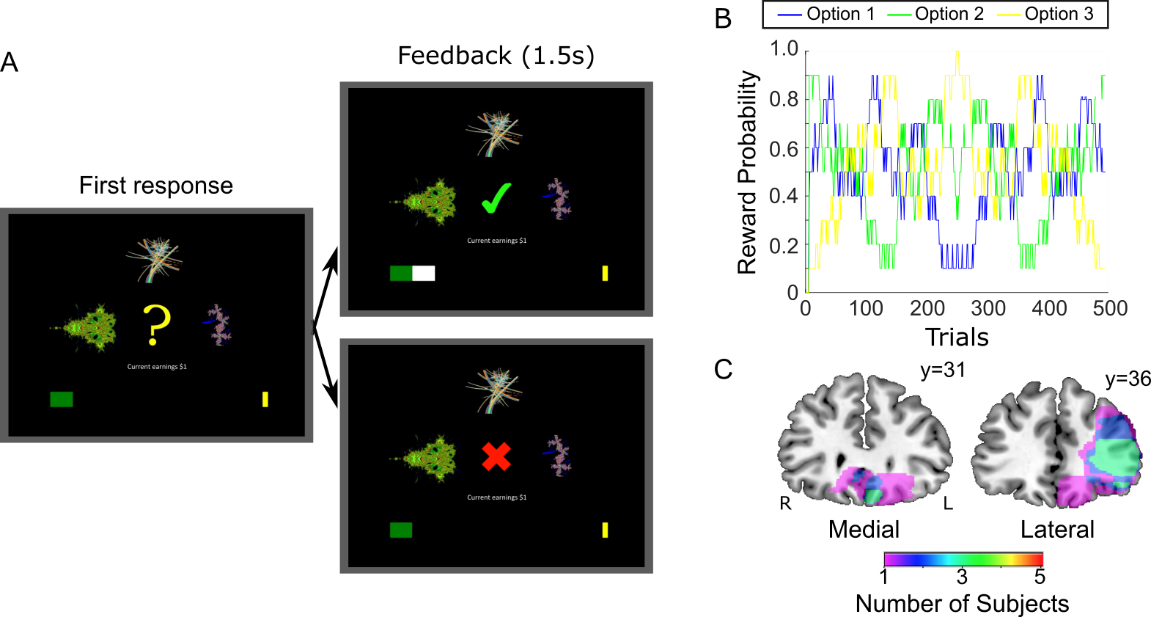
**

**Fig F. Study 2: Task design, reward schedule and lesion overlap in human patients.** **(A)** Trial timeline: In each testing session, human patients made choices among three novel stimuli (fractal images; left-hand side) via a keyboard button press response. Visual feedback of choice was delivered according to the particular reward schedule (right-hand side). Both possible outcomes are displayed in this example: a green tick was delivered in the case of a positive outcome (top panel) and a red cross during no reward events (bottom panel). **(B)** Reward schedules comprised three options whose reward probabilities ranged between 0.1 and 1 and drifted throughout the session, with each option being competitive at some time during the session (i.e. each option was the best one at least during a short phase of the session). Participants performed this task twice using the same reward schedule but different stimuli. **(C)** Medial (Left) and lateral frontal lobe (right) lesion outlines as based on patients' most recent scan represented on the MNI standard template. Colorbar indicates lesion overlap (n=4 and n=4 respectively).


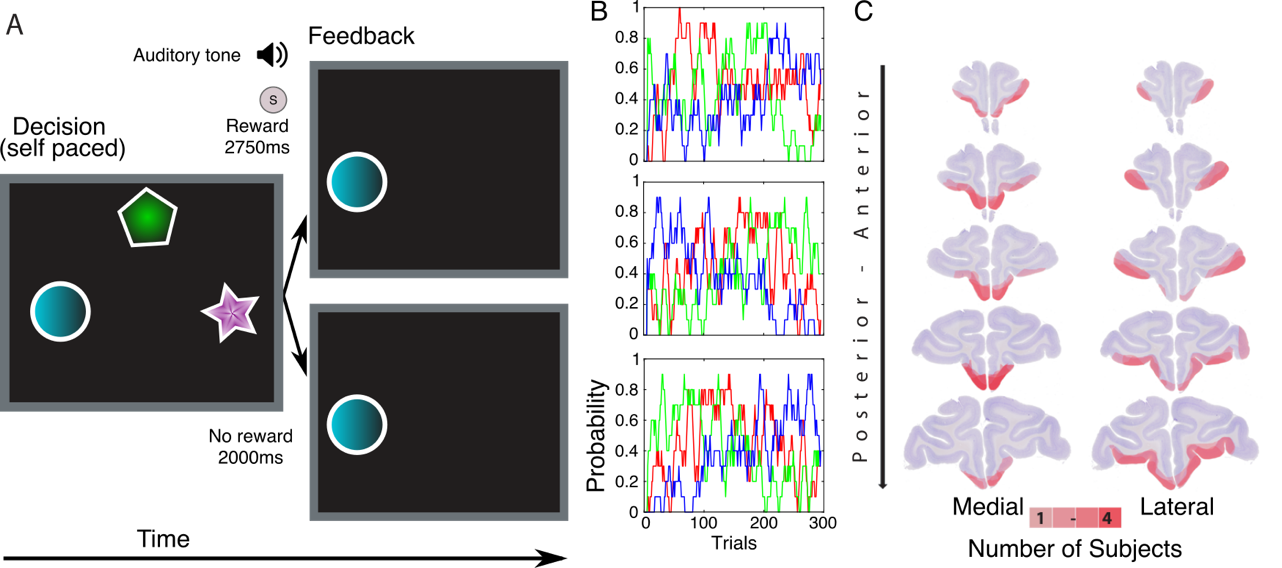


**Fig G. Study 3: Task design, reward schedule and lesion.** **(A)** Trial timeline: In each testing session, macaques made choices among three novel stimuli (novel clip art images; left-hand side) via a touch screen before receiving auditory feedback and, according to the particular reward schedule, a sucrose pellet (S) reward or nothing (right-hand side). The chosen stimuli remained onscreen during feedback. Both possible outcomes are displayed in this example: A reward pellet and auditory feedback was delivered in the case of a positive outcome (top panel) and nothing happened during no reward events (bottom panel). **(B)** Animals made choices across three similar reward schedules in which the reward probabilities ranged between 0 and 1 and drifted throughout the session, with each option being competitive at some time during the session (i.e. each option was the best one at least during a short phase of the session). **(C)** Medial (left) and lateral frontal lobe (right) lesion locations represented on an unoperated control, with redness indicating lesion overlap (n=4 and n=3 respectively).

**Table A. Summary Table of Pearson R values, R^2^ values and AIC values for linear and quadratic model fits**. Table shows for key behavioural (percentages or beta weights) and neural (gray matter Jacobean values) results related to age. In all instances the coefficients of linear or quadratic polynomial fits were compared using a likelihood-ratio test. The best fitting model was indexed by the lowest AIC value. The last row summarizes the winning model (linear or quadratic) for the key behavioural analyses and five GM regions of interest; lOFC (lateral orbitofrontal cortex), mOFC (medial orbitofrontal /ventromedial prefrontal cortex), amygdala, dACC (dorsal anterior cingulate) and ains (anterior insula). * p<0.05.

| **Factors** | Pearson R | Linear R^2^ | Quadratic R^2^ | AIC linear | AIC quadratic | AIC min |
| --- | --- | --- | --- | --- | --- | --- |
| Total rewards (Fig.2) | 0.160* | 0.026 | 0.031 | 2.503 | 2.502 | Quad |
| % Best choice (Fig.2) | 0.290* | 0.086 | 0.11 | -0.336 | -0.345 | Quad |
| CxR_t_ (Fig.3) | 0.220* | 0.05 | 0.049 | -0.534 | -0.533 | Lin |
| GRS (Fig.3) | -0.140* | 0.018 | 0.018 | -0.934 | -0.933 | Lin |
| lOFC (Fig.S5) | -0.470* | 0.223 | 0.28 | -381.518 | -388.294 | Quad |
| mOFC (Fig.S5) | -0.210* | 0.045 | 0.101 | -381.574 | -390.139 | Quad |
| Amygdala (Fig.S5) | 0.030 | 0.001 | 0.03 | -397.312 | -400.072 | Quad |
| dACC (Fig.S5) | 0.050 | 0.003 | 0.03 | -418.289 | -416.039 | Lin |
| ains  (Fig.S5) | **-**0.390* | 0.149 | 0.171 | -299.374 | -301.559 | Quad |

Supplementary Methods for Fig E

*Developmental neuroanatomy of medial and lateral prefrontal cortex*

Participants

In parallel to Study 1, 125 individuals, aged 11-35 years, were sub-sampled from the Human Connectome Project (HCP) Young Adult (HCPYA, *n*=56) and HCP Developmental (HCPD, *n*=69) databases [13]. Informed by societal and neuroanatomical developmental changes [20,21]and the late maturation of the prefrontal cortex [18], participants were categorized into three age groups, 11-17, 18-23 and 24-35 years old. Roughly equally sized populations were sampled within each age bracket from the databases to contribute to the non-biased study specific grey matter (GM) template from the pre-processed T1 weighted (T1w) images available at the time of analysis (11-17 years; n=43 from HCPD, 18-23 years; n=26 from HCPD and n=13 from HCPYA, 24-35 years; n=43 from HCPYA).

Image Acquisition, Pre-Processing and Statistical Analysis

T1w structural images were acquired with HCP acquisition parameters [22]: 256 sagittal slices were acquired with TR = 2400ms, TE = 2.14ms, flip angle = 8°, FOV = 224mm, matrix = 320. The primary aim of these selected parameters was to achieve high spatial resolution: T1w images had a voxel size of 0.7mm isotropic. All structural images underwent minimal pre-processing via the three HCP structural pipelines *PreFreeSurfer*, *FreeSurfer*, and *PostFreeSurfer*. The *PreFreeSurfer* pipeline generates a *Native Volume Space* brain-extracted, undistorted T1w image which is linearly aligned to the axes of MNI space. Full details of structural image acquisition and minimal pre-processing pipelines are reported by Glasser et al [22].

Previous studies showed that prefrontal cortex, as a whole, matures relatively late, well into early adulthood [18,19]. Designed to complement the behavioural developmental study from the outset we sought to identify neural maturation at a resolution on par with current state-of-the art functional localization methods. To that end we used connectivity-based parcellation of frontal cortex [15] and the probabilistic Harvard-Oxford atlas [23–26]. This allowed us to delineate neural maturation specific to key nodes within a learning and decision-making network: lateral orbitofrontal cortex, medial orbitofrontal/ventromedial prefrontal cortex, amygdala and anterior cingulate cortex [27]. Lateral orbitofrontal and medial orbitofrontal/ventromedial prefrontal cortex are parts of distinct structural networks [28,29], exhibit distinct functional connectivity patterns [15] and have different functional specializations. Lesion studies have strongly linked local reward learning to lateral orbitofrontal cortex in macaques [3–5], while macaque neuroimaging identified global reward signals in closely adjacent posterior-lateral regions [1], making lateral orbitofrontal cortex a key candidate region to reflect our observed developmental changes in terms of grey matter maturation. We focus here on areas 47/12o and 14 within lateral and medial orbitofrontal cortex respectively as lesions in this area casually disrupt the local reward learning mechanism and the bias by irrelevant alternative mechanism respectively [2,3]. We also include a characterisation of the grey matter (GM) trajectory of the amygdala, a subcortical region known to be heavily structurally and functionally interconnected with lateral orbitofrontal cortex, and known to have a complementary role in value guided learning [10,11,30,31], the anterior cingulate cortex, a region with a critical role in, among other functions, learning from feedback and prediction errors [32,33] and the anterior insula, where fMRI signals tracking the global reward state have been identified in macaques [1].

To identify fine scale differences in GM maturation between these regions we adapted the FSL voxel-based morphometry (VBM) analysis pipeline [34]. First, the segmented HCP-provided skull stripped T1w images were affine-registered to the GM ICBM-152 template using FLIRT [35], followed by nonlinear registration using FMRIB’s Nonlinear Image Registration Tool (FNIRT) [36]. Next, a randomly selected sub-sample of the resulting images were averaged to compose the non-bias study-specific template (*n* = 39 from each age group, referred to as template images), flipped along the x-axis and averaged again, creating a symmetric template. The final symmetric template was produced by non-linearly re-registering individual subject GM template images to symmetric template, flipping along the x-axis, and again averaging. Finally, the native GM images of all participants were non-linearly re-registered to this template and concatenated into a 4D image. The resulting unsmoothed 4D GM image was modulated for the contraction or enlargement of each subject’s image to the template with each voxel of each image being multiplied by the Jacobian of the warp field [37]. No additional smoothing was implemented. From this point we extracted all participants’ values from the 4D GM modulation image – from here on these values will be referred to as “GMm” - for our medial and lateral orbitofrontal cortex (PFC) region of interest (ROI) mask. Higher GMm values reflect regional expansion of the native GM images relative to the template and thus likely correspond to more GM. By contrast, lower values reflect regional GM contraction in native images and therefore likely correspond to less GM. Variance associated with sex, handedness and total brain volume were regressed out of GMm for each ROI using MATLAB (2014a, The Mathworks, Inc., Natick, MA, United States). The resulting residual GMm were used in analyses.

Frontal subregions were selected a-priori on the basis of Neubert et al.’s [15] connectivity-based parcellation of the human medial and orbital regions of the prefrontal cortex. From this parcellation we selected the clusters corresponding to area 47/12o (lateral orbitofrontal cortex), areas 14 (medial orbitofrontal cortex), area RCZa (dorsal anterior cingulate cortex). The bilateral amygdala and anterior insula masks was based on the Harvard-Oxford atlas.

To investigate and compare GM trajectories between lateral orbitofrontal and the other brain regions we first compared developmental trajectories across all five regions [ROI: lateral | ins | medial | amygdala | dACC) x age] ANCOVA. This was followed up with analyses focused at comparing lateral orbitofrontal cortex directly with the other four regions directly [ROI: lateral v ins | lateral v medial | lateral v amygdala | lateral v ACC) x age]. All key assumptions necessary to subject the data to a repeated measures ANCOVA were tested and met. With both the dependent variable, GMm, and the covariate, age, measured on continuous scales, and the independent variables being subregions of PFC and hemispheres. No outliers were identified (>4 standard deviations from the mean). Residuals were approximately normally distributed for each category of the independent variable (Shapiro-Wilk tests p > 0.05). Homoscedasticity was confirmed via scatterplots of the standardized residuals against the predicted values and all coefficient VIF collinearity statistics reported as <10.

Residuals were subjected to ANCOVA methodology adapted from Schneider and colleagues [38]. Age, the covariate, was de-meaned across all of the participants before being entered into repeated measures ANCOVAs. We examined evidence in favour of the hypothesis of a differential effect of age on ROI GMm (i.e., an interaction between ROI GMm and age). Schneider et al [38] then advise employing a standard ANOVA (2(ROI) x 2(hemisphere)) to examine any effects not involving the age covariate. However, because the study-specific GM template is an age-wise representative average of the brain structure *and* the GMm values are de-meaned when estimating the residuals (removing the variance of handedness, sex and total brain volume) the mean of each ROI is zero. Therefore, we anticipate no independent main effects of PFC subregion or hemisphere. Instead, our primary focus is the *relative* differences in GM trajectories, not differences in absolute GM between brain regions. Accordingly, we do not report the follow-up ANOVA, but only focus on interactions involving age.

As a follow-up analysis we further characterised the trajectory of GM maturation by fitting linear and quadratic link functions. Among these functions, we identified the one with the best fit as indicated by the lowest AIC value and reported Table A in S1 Text.

**References:**

1. Wittmann MK, Fouragnan E, Folloni D, Klein-Flügge MC, Chau BKH, Khamassi M, et al. Global reward state affects learning and activity in raphe nucleus and anterior insula in monkeys. Nat Commun. 2020;11: 3771. doi:10.1038/s41467-020-17343-w

2. Noonan MP, Chau B, Rushworth MF, Fellows LK. Contrasting effects of medial and lateral orbitofrontal cortex lesions on credit assignment and decision making in humans. J Neurosci. 2017. doi:10.1523/JNEUROSCI.0692-17.2017

3. Noonan MP, Walton ME, Behrens TE, Sallet J, Buckley MJ, Rushworth MF. Separate value comparison and learning mechanisms in macaque medial and lateral orbitofrontal cortex. Proc Natl Acad Sci U S A. 2010;107: 20547–52. doi:10.1073/pnas.1012246107

4. Rudebeck PH, Saunders RC, Prescott AT, Chau LS, Murray EA. Prefrontal mechanisms of behavioral flexibility, emotion regulation and value updating. Nat Neurosci. 2013;16: 1140–5. doi:10.1038/nn.3440

5. Walton MEM, Behrens TEJT, Buckley MMJ, Rudebeck PH, Matthew FS, Rushworth MFS. Separable learning systems in the macaque brain and the role of orbitofrontal cortex in contingent learning. Neuron. 2010;65: 927–39.

6. Chau BK, Law C-K, Lopez-Persem A, Klein-Flügge MC, Rushworth MF. Consistent patterns of distractor effects during decision making. eLife. 2020;9: e53850. doi:10.7554/eLife.53850

7. Hunt LT, Kolling N, Soltani A, Woolrich MW, Rushworth MF, Behrens TE. Mechanisms underlying cortical activity during value-guided choice. Nat Neurosci. 2012;15: 470–6, S1-3. doi:10.1038/nn.3017

8. Meder D, Kolling N, Verhagen L, Wittmann MK, Scholl J, Madsen KH, et al. Simultaneous representation of a spectrum of dynamically changing value estimates during decision making. Nat Commun. 2017;8: 1942. doi:10.1038/s41467-017-02169-w

9. Sallet J, Noonan MP, Thomas A, O’Reilly JX, Anderson J, Papageorgiou GK, et al. Behavioral flexibility is associated with changes in structure and function distributed across a frontal cortical network in macaques. Ashe J, editor. PLoS Biol. 2020;18: e3000605. doi:10.1371/journal.pbio.3000605

10. Murray EA, Fellows LK. Prefrontal cortex interactions with the amygdala in primates. Neuropsychopharmacol. 2022;47: 163–179. doi:10.1038/s41386-021-01128-w

11. Chau BK, Sallet J, Papageorgiou GK, Noonan MP, Bell AH, Walton ME, et al. Contrasting Roles for Orbitofrontal Cortex and Amygdala in Credit Assignment and Learning in Macaques. Neuron. 2015;87: 1106–18. doi:10.1016/j.neuron.2015.08.018

12. Klein-Flügge MC, Wittmann MK, Shpektor A, Jensen DEA, Rushworth MFS. Multiple associative structures created by reinforcement and incidental statistical learning mechanisms. Nat Commun. 2019;10: 4835. doi:10.1038/s41467-019-12557-z

13. Somerville LH, Bookheimer SY, Buckner RL, Burgess GC, Curtiss SW, Dapretto M, et al. The Lifespan Human Connectome Project in Development: A large-scale study of brain connectivity development in 5–21 year olds. NeuroImage. 2018;183: 456–468. doi:10.1016/j.neuroimage.2018.08.050

14. Harms MP, Somerville LH, Ances BM, Andersson J, Barch DM, Bastiani M, et al. Extending the Human Connectome Project across ages: Imaging protocols for the Lifespan Development and Aging projects. NeuroImage. 2018;183: 972–984. doi:10.1016/j.neuroimage.2018.09.060

15. Neubert FX, Mars RB, Sallet J, Rushworth MF. Connectivity reveals relationship of brain areas for reward-guided learning and decision making in human and monkey frontal cortex. Proceedings of the National Academy of Sciences of the United States of America. 2015;112: E2695-704. doi:10.1073/pnas.1410767112

16. Dumontheil I. Adolescent brain development. Current Opinion in Behavioral Sciences. 2016;10: 39–44. doi:10.1016/j.cobeha.2016.04.012

17. Mills KL, Goddings A-L, Clasen LS, Giedd JN, Blakemore S-J. The Developmental Mismatch in Structural Brain Maturation during Adolescence. Dev Neurosci. 2014;36: 147–160. doi:10.1159/000362328

18. Gogtay N, Giedd JN, Lusk L, Hayashi KM, Greenstein D, Vaituzis AC, et al. Dynamic mapping of human cortical development during childhood through early adulthood. PNAS. 2004;101: 8174–8179. doi:10.1073/pnas.0402680101

19. Raznahan A, Lee Y, Stidd R, Long R, Greenstein D, Clasen L, et al. Longitudinally mapping the influence of sex and androgen signaling on the dynamics of human cortical maturation in adolescence. Proceedings of the National Academy of Sciences. 2010;107: 16988–16993. doi:10.1073/pnas.1006025107

20. van Duijvenvoorde ACK, Peters S, Braams BR, Crone EA. What motivates adolescents? Neural responses to rewards and their influence on adolescents’ risk taking, learning, and cognitive control. Neuroscience & Biobehavioral Reviews. 2016;70: 135–147. doi:10.1016/j.neubiorev.2016.06.037

21. Viner RM, Ozer EM, Denny S, Marmot M, Resnick M, Fatusi A, et al. Adolescence and the social determinants of health. The Lancet. 2012;379: 1641–1652. doi:10.1016/S0140-6736(12)60149-4

22. Glasser MF, Sotiropoulos SN, Wilson JA, Coalson TS, Fischl B, Andersson JL, et al. The minimal preprocessing pipelines for the Human Connectome Project. NeuroImage. 2013;80: 105–124. doi:10.1016/j.neuroimage.2013.04.127

23. Makris N, Goldstein JM, Kennedy D, Hodge SM, Caviness VS, Faraone SV, et al. Decreased volume of left and total anterior insular lobule in schizophrenia. Schizophrenia Research. 2006;83: 155–171. doi:10.1016/j.schres.2005.11.020

24. Frazier JA, Chiu S, Breeze JL, Makris N, Lange N, Kennedy DN, et al. Structural Brain Magnetic Resonance Imaging of Limbic and Thalamic Volumes in Pediatric Bipolar Disorder. AJP. 2005;162: 1256–1265. doi:10.1176/appi.ajp.162.7.1256

25. Goldstein JM, Seidman LJ, Makris N, Ahern T, O’Brien LM, Caviness VSJ, et al. Hypothalamic abnormalities in schizophrenia: sex effects and genetic vulnerability. Biol Psychiatry. 2007;61: 935–945. doi:10.1016/j.biopsych.2006.06.027

26. Desikan RS, Ségonne F, Fischl B, Quinn BT, Dickerson BC, Blacker D, et al. An automated labeling system for subdividing the human cerebral cortex on MRI scans into gyral based regions of interest. NeuroImage. 2006;31: 968–980. doi:10.1016/j.neuroimage.2006.01.021

27. Rushworth MF, Noonan MP, Boorman ED, Walton ME, Behrens TE. Frontal cortex and reward-guided learning and decision-making. Neuron. 2011;70: 1054–69. doi:10.1016/j.neuron.2011.05.014

28. Ongur D, Price JL. The organization of networks within the orbital and medial prefrontal cortex of rats, monkeys and humans. Cereb Cortex. 2000;10: 206–219. doi:10.1093/cercor/10.3.206

29. Petrides M, Tomaiuolo F, Yeterian EH, Pandya DN. The prefrontal cortex: comparative architectonic organization in the human and the macaque monkey brains. Cortex. 2012;48: 46–57. doi:10.1016/j.cortex.2011.07.002

30. Rudebeck PH, Ripple JA, Mitz AR, Averbeck BB, Murray EA. Amygdala contributions to stimulus-reward encoding in the macaque medial and orbital frontal cortex during learning. J Neurosci. 2017. doi:10.1523/JNEUROSCI.0933-16.2017

31. Rudebeck PH, Mitz AR, Chacko RV, Murray EA. Effects of amygdala lesions on reward-value coding in orbital and medial prefrontal cortex. Neuron. 2013;80: 1519–31. doi:10.1016/j.neuron.2013.09.036

32. Behrens TEJ, Woolrich MW, Walton ME, Rushworth MFS. Learning the value of information in an uncertain world. Nat Neurosci. 2007;10: 1214–21.

33. Noonan MP, Mars RB, Rushworth MF. Distinct roles of three frontal cortical areas in reward-guided behavior. The Journal of neuroscience : the official journal of the Society for Neuroscience. 2011;31: 14399–412. doi:10.1523/JNEUROSCI.6456-10.2011

34. Douaud G, Smith S, Jenkinson M, Behrens T, Johansen-Berg H, Vickers J, et al. Anatomically related grey and white matter abnormalities in adolescent-onset schizophrenia. Brain. 2007;130: 2375–2386. doi:10.1093/brain/awm184

35. Jenkinson M, Smith S. A global optimisation method for robust affine registration of brain images. Medical Image Analysis. 2001;5: 143–156. doi:10.1016/S1361-8415(01)00036-6

36. Jenkinson M, Beckmann CF, Behrens TEJ, Woolrich MW, Smith SM. FSL. NeuroImage. 2012;62: 782–790. doi:10.1016/j.neuroimage.2011.09.015

37. Good CD, Johnsrude IS, Ashburner J, Henson RNA, Friston KJ, Frackowiak RSJ. A Voxel-Based Morphometric Study of Ageing in 465 Normal Adult Human Brains. NeuroImage. 2001;14: 21–36. doi:10.1006/nimg.2001.0786

38. Schneider BA, Avivi-Reich M, Mozuraitis M. A cautionary note on the use of the Analysis of Covariance (ANCOVA) in classification designs with and without within-subject factors. Front Psychol. 2015;6. doi:10.3389/fpsyg.2015.00474
