# Supplementary material for: Local and global reward learning in the lateral frontal cortex show differential development during human adolescence
Source: PLoS Biol. 2023 Mar 2;21(3):e3002010. doi: 10.1371/journal.pbio.3002010 (PMC10013901; doi:10.1371/journal.pbio.3002010)
Supplement: Attachment — Submitted filename: ResponsetoReviewers_submit.docx [file pbio.3002010.s003.docx]

**Response to the reviewers.**

**Reviewer 1:**

**To strengthen the theoretical claims and conceptual coherence of this excellent manuscript, I would recommend the following revisions.**

*We thank the reviewer for their very supportive comments. We have addressed their points below and have highlighted changes in our manuscript that we made in response to the reviewer’s comments in red.*

**1. Please describe in further detail the relationship(s) between the different pieces of evidence presented in this work, focusing on their integration and the potential methodological limitations this effort faces.**

*Thank you for raising this point. We have now expanded the integration of the different studies in each section of the manuscript: abstract, introduction, results, and discussion.* *We have also followed the advice of the other two reviewers and moved the structural analysis of the Human Connectome project data into the supplements. This analysis showed greatest grey matter maturation in lateral compared to medial prefrontal cortex. The methodological limitation of this study was that it was not conducted in the same developmental sample as the behavioural developmental study – which we clearly acknowledge now as explained below. We included instead a reanalysis of previously published macaque data (current study 3). We referred to this data before in our introduction. The macaque work was foundational for the current study and by reanalysing it with the same analysis pipeline as the other studies allows us to relate our work even more directly to the macaque work. We have also made a conceptual figure that summarises the effects of the global reward state (GRS) effects we observed across the three studies (study 1: developmental study; study 2: human lateral lesion study; study 3: macaque lateral lesion study).*

*In the manuscript, we have improved the integration of the studies in the following way:*

***Revised abstract:***

Reward-guided choice is fundamental for adaptive behaviour and depends on several component processes supported by prefrontal cortex. Here, across three studies, we show that two such component processes, linking reward to specific choices and estimating the global reward state, develop during human adolescence and are linked to the lateral portions of the prefrontal cortex. These processes reflect the assignment of rewards contingently to local choices, or non-contingently, to choices that make up the global reward history. Using matched experimental tasks and analysis platforms we show the influence of both mechanisms increase during adolescence (study 1), and that lesions to lateral frontal cortex (that included and/or disconnected both orbitofrontal and insular cortex) in human adult patients (study 2) and macaque monkeys (study 3) impair both local and global reward learning. Developmental effects were distinguishable from the influence of a decision bias on choice behaviour, known to depend on medial prefrontal cortex. Differences in local and global assignments of reward to choices across adolescence, in the context of delayed GM maturation of the lateral orbitofrontal and anterior insula cortex, may underly changes in adaptive behaviour.

*Moreover, we have now clarified the links between the studies in the Introduction and the Discussion. This is the key part of the Introduction explaining the relationships between the studies.* ***Introduction, page 5-6:***

Here, we combine behavioural and lesion investigations to suggest an important role for neighbouring subregions of the lateral frontal cortex, specifically orbitofrontal and anterior insular cortex in the development of local and global reward learning. We used the same multi-option probabilistic learning task originally developed in macaques to dissociate local and global reward learning (Fig.1) [4,6,14]. In study 1, we tested a large online sample (overall *n*=422) of adolescents (11-17 years) and young adults (age 18-35 years) and show that both local and global reward learning change during human adolescence. We chose this age range in accord with previous work [47] and in particular in reference to the protracted maturation profile of lateral prefrontal cortex [32]. Our findings suggest that young adults associated choices more strongly with local rewards and were simultaneously negatively influenced by the GRS. The GRS influence became even more negative with age, meaning young adults, more than adolescents, contextualized their choices within the longer-term reward context, and were less likely to persist with a choice if the alternatives afforded by this context were attractive. By contrast, decision bias mechanisms that depend on the ventromedial portions of the orbitofrontal cortex showed no relationship with age.

The finding that the behavioral mechanisms of local and reward learning continue to change during adolescence align well with the non-human primate literature [6,7,14,17] and our knowledge about structural brain maturation in humans: lateral frontal, compared to medial frontal brain regions, appear to continue to mature during adolescence well into adulthood [30,33,34] and hence we would expect functions that depend on this part of the brain to keep changing during this time period as well. However, only manipulation approaches can provide evidence for a causal reliance of a cognitive function on a neural substrate. Study 2 therefore examined the impact of broad lesions to lateral frontal cortex (lesions included and/or disconnected both orbitofrontal and insular cortex) on local and global reward learning. These studies used experimental tasks and analyses pipelines that were tightly matched to study 1 in cohorts of adult patients with medial or lateral frontal lobe lesions. The results indicated that indeed intact lateral frontal cortex is causally important for both local and global reward learning. Finally, in Study 3, we reanalyse non-human primate data [6,14] that has initially suggested that these lateral frontal regions are important for local reward learning, again using matched experimental tasks and analysis pipelines. This uncovered that lateral lesions in macaques (that likely disconnected both orbitofrontal and insular cortex), too, impair global reward learning as well and give new insights into how the GRS guides choices differently in humans and macaques. While humans showed negative GRS effects, macaques showed positive ones. Together, our results suggest that local and global reward learning mature during adolescence (study 1) and that both learning mechanisms causally depend on (subregions within) lateral frontal cortex (study 2,3). This suggests that the protracted neural maturation in lateral frontal regions [30,33,34] is a key driver for the maturation of local and global reward learning during adolescence.

*In addition, we include the following figure to illustrate the conceptual relationships of the identified GRS effects across studies.* ***Figure 6, page 19:***

**Figure 6. Conceptual summary of GRS effects across studies. Study 1: Upper two panels.** People pursue a current choice (banana symbol within circle). They also experience the GRS, the global levels of reward afforded by the environment over time (tree symbol on the periphery of the circle). Adolescents switch away from the currently pursued choice if the GRS is high (small arrow pointing outwards). Adults do this contrast effect of the GRS even more strongly. They contextualise the current choice within the set of alternative options and the knowledge that rich alternatives exist makes adults switch away from their currently pursued course of action. This means that the GRS exerts a ‘contrast effect’: current choices appear less valuable if the GRS is very high. The increased reliance on the GRS coincides with grey matter maturation in lateral frontal lobe regions including the anterior insula and lateral orbitofrontal cortex. **Study 2:** **Lower left panel:** Lesions to lateral frontal lobe (affecting multiple subregions) reduces the GRS effect in human adults. **Study 3:** **Lower right panel:** Macaques also contextualise current choices within the GRS. In contrast to humans, macaques use the GRS fundamentally differently. They show ‘spread of effect’: The GRS positively affects the value of a current choice and this makes macaques stay more with a choice if the GRS is high. Strikingly, lesions to lateral frontal lobe (again affecting multiple subregions) *increase*, rather than decrease, the GRS effect in macaques (thick arrow surrounding small arrow indicates stronger GRS effect after lesion to lateral frontal lobe).

*In the* ***Discussion, page 20*** *we again highlight links across the studies****:***

**Discussion**

We investigated the development of component processes of reward learning that have been linked to neighbouring regions of orbitofrontal and anterior insula cortex in studies of non-human primates: local reward learning (or ‘contingent credit assignment’) and non-contingent global reward learning based on the GRS [6–8,14,17,18]. These reward-related brain regions have a particularly protracted maturation profile and continue to change well into adulthood [30,33,34] (Fig.S5). Therefore, we tested whether cognitive functions that are likely to depend on these regions keep changing during this time period as well. Indeed, we have shown that both local and global reward learning matured during development. We showed that participants’ decision to switch or stay with the current choice was positively influenced by local reward that was received for making a specific choice and negatively influenced by the GRS, and these mechanisms increased in their respective influence across adolescent development (study 1; Fig.3). In contrast, we found that reward-guided decision mechanisms linked to more medial frontal lobe regions did not show developmental differences over the same age range (Fig.S1,S2). However, only manipulation experiments such as lesion studies can reveal a causal relationship between a neural substrate and a cognitive process. We conducted two lesion studies – one in humans (study 2) and one in macaques (study 3) – that assessed the impact of lesions to broad parts of lateral frontal cortex (likely affecting both the anterior insula and lateral orbitofrontal cortex, see below) to local and global reward learning. The experimental paradigm was a 3-armed bandit task (Fig.1) and was closely matched across studies. We used the same “credit assignment GLM” [7] in all studies to ensure that all three studies measured local and global reward learning in the same way. Both lesion studies showed that lateral prefrontal cortex is indeed causally necessary for intact local and global reward learning in both species (Fig.4,5). This suggest that structural changes in lateral parts of prefrontal cortex underlie the developmental changes we observed in behaviour. Strikingly, humans and macaques differed in the way they were guided by the GRS. Humans used the GRS to ‘contrast’ it with the current choice and were likely to switch away from a choice if the GRS was high [50,54,60,61]; macaques showed ‘spread of effect’ [6,11,15] and were more likely to stay with their choices if the GRS was high. Lesions to lateral frontal cortex altered the GRS effect in both species. However, while it abolished the negative GRS effect in humans, in increased the positive GRS effect in macaques (Fig.6).

*Moreover, we have added a discussion of the limitations of our multi-pronged approach to the* ***discussion****:*

***1. Page 24 and 26****, on the grey matter study conducted in a separate sample than the behavioural study:*

Longitudinal studies examining grey matter maturation and the development of reward learning in the same sample are needed to provide more direct evidence about this link between cognitive and neural development.

[…]

Future longitudinal studies should examine multimodal changes in lateral orbitofrontal and anterior insula cortex and the respective parallel changes in the adaptive influence of local and global reward learning.

***2. Page 25****, on interpreting species differences:*

This potential species difference is striking, particularly considering that we used a variant of a widely used probabilistic learning task that was matched across studies. However, species comparisons are inherently difficult to interpret. For example, despite the matched tasks, clear differences persisted in the way subjects were introduced to the study (verbal instructions versus weeks of training) and the setting in which the experiments were conducted.

**2. Please include citations to the human lesion literature examining lateral prefrontal cortex lesions and their roles in intelligence, fluid cognition, working memory, and/or other facets of intelligence that bear on the present study (e.g., by Duncan, Grafman, and others).**

*We thank the reviewer for this suggestion and now reflect on the importance of understanding the interactions between the development of the cognitions dependent on the late developing dorsolateral prefrontal cortex and those mechanisms examined in the current study that depend on the lateral orbitofrontal cortex. We have added this and the suggested citations to the Discussion of our results accordingly.* ***Page 26 Discussion:***

Lateral prefrontal regions, beyond orbitofrontal cortex, are also all late to mature across adolescence [30,89]. The more dorsolateral prefrontal regions are associated with intelligence, fluid cognition, working memory and attentional control [90–92]. A critical direction for future work will be to examine the interactions between these developing cognitions, and brain network dynamics, and the learning mechanisms described here. Recent advances in network neuroscience offer exciting methods to characterise individual differences in complex cognitions as a function of local and global brain network topology and community structure [93–95]. Characterising these interactions could ultimately improve predictions of transdiagnostic features of neurodevelopmental and behavioural trajectories.

**3. It would also be valuable to discuss recent work in network neuroscience that examines the role of local and global brain network efficiency in intelligence (e.g., by Barbey, Sporns, and others).**

*We agree with the reviewer that network neuroscience approaches offer complementary methods that can deepen our understanding of the contribution of brain dynamics to the individual differences in the development of complex cognition and behaviour. We now also include these reflections in the same part of the Discussion as the last point and cite the suggested authors (see response to point 2 and relevant section in the Discussion on page 26).*

**4. Please describe in greater detail the theoretical claims supported by the present findings and their contribution(s) to the larger literature.**

*We have made substantial revisions to the discussion in light of this reviewers (and others) suggestions in which we argue that one of the findings from this study is that, across adolescence, humans are increasingly influenced by local and global reward states to support adaptive behaviour. We argue that the mechanisms are causally affected by lesions that damage the lateral orbitofrontal cortex (and likely disconnect the anterior insula) and that these two structures show protracted gray matter maturation relative to other known learning and decision making areas in the brain. Each portion of these claims are discussed in detail and contextualised in the literature. New sections of the discussion include:*

***Page 22/23:***

Our results contribute to the debate about the development of reinforcement learning during development [9,44]. Studies indicate that overall the learning rate, i.e. the speed of updating the value of a choice, increases during adolescence [41–43,43–46]. We find the same in our study 1 (Fig.S2). Moreover, the increase in local reward learning in our “credit assignment GLM” could be interpretated along similar lines – as an increase of the weight that an outcome has on changing an option’s value. The strong positive correlation between the learning rate from our reinforcement learning model and local reward learning effect size is a further indication of this. However, studies have started to examine increases in reward learning rate in more detail. As highlighted above, the precise task context plays a big role in whether increased learning rates are observed and if they are desirable to optimise rewards [42]. Another consideration is that learning from outcomes might differ depending on whether that outcome is positive or negative, although these effects, again, appear context-dependent [43,45,83,84]. Our findings that the GRS exerts an increasingly negative effect during development adds to these ideas and highlight influences on reward learning that go beyond changes in a unitary reward learning rate. GRS effects were unrelated to a simple reward learning rate in previous work [7] and also in our current data set. Instead, they contextualise a current choice based on the global reward environment. This mechanism can add to the changes in value observed for a choice and can, in effect, lead to different effective learning rates for positive and negative outcomes [7,51,52]. The negative GRS effects observed here predict higher learning rates for positive outcomes if the GRS is low, and higher learning rates for negative outcomes if the GRS is high. A promising avenue for future research would be to follow-up on these predictions and conduct a more formal modelling analysis of the developmental GRS effects. It might help explain diverging results by suggesting that analyses of reward learning rates should take into account the global reward levels present in the experiments. Neurally, our results suggest that subregions within lateral frontal cortex, specifically anterior insula and orbitofrontal cortex, are particularly promising target regions to look for neural correlates of the development of reinforcement learning mechanisms. These subregions have been shown to integrate rewards with different time constants in adults [7,60,80] and, in adolescents, higher learning rates to negative prediction errors are linked to greater activity in the anterior insula [43].

***Page 25:***

However, even longitudinal studies are usually correlative and as such can only provide limited evidence about causal relationships. Study 2 and 3 therefore used finely matched experimental paradigms and analysis techniques to directly assess the causal importance of lateral frontal regions in local and global reward learning. In humans we show that the GRS effect is reduced (i.e. closer to zero) after lateral prefrontal lesions, compared to medial orbitofrontal lesions, in combination with decreased local reward learning (Fig.4). Note that the direction of change in both local and global reward learning after lesions is highly compatible with the correlation between those two variables in our online sample (Fig.S3). This suggests that both learning processes are at least partly supported by neural mechanisms in lateral frontal cortex. In study 3, we confirm in macaque monkeys that the GRS effect is altered after lateral lesions (Fig.5). The macaque lesion effects, however, were qualitatively different. First, as we know from the same data in past work [6,14], local reward learning is reduced after lateral lesions. This means that both humans and macaques show a decrease of contingent/local reward learning after broad lesions to lateral prefrontal cortex. However, rather than decreasing the GRS effect as in humans, lateral lesions *increased* the GRS effect in macaques. While a negative GRS effect was abolished in humans, a positive GRS effect was increased in macaques (Fig.6). Together with the decline of local reward learning in both species, this leads to the contention that the GRS effect reflects a task-adaptive process in humans, that matures during adolescences and is compromised by lesions, whereas in monkeys, the GRS effect reflects a suboptimal ‘spread’ of reward which is even increased by lateral frontal lobe lesions.

In all analysed macaque data sets here and consistent with previous work on the GRS [7] and credit assignment [6,17], in macaques, the effect of the GRS on choice was *positive*. This contrasted with the negative GRS effect in human participants (study 1,2). This potential species difference is striking, particularly considering that we used a variant of a widely used probabilistic learning task that was matched across studies. However, species comparisons are inherently difficult to interpret. For example, despite the matched tasks, clear differences persisted in the way subjects were introduced to the study (verbal instructions versus weeks of training) and the setting in which the experiments were conducted. Nevertheless, one interpretation of the observed GRS differences is that human behaviour was more in line with ideas from optimal foraging theory that suggest that a value’s choice should be contrasted with the background reward rate of the environment [49,50,54,60,87]. This can promote optimal choice switching and exploration [51,52,54]. However, this view assumes that participants treated the trials in the experiment as discrete, unrelated instances. In contrast, the positive GRS effect in macaques might suggest that non-human primates do not perceive the task as a series of discrete and unrelated trials. Instead, they might expect inter-trial contingencies, for example, that an action on trial n has an influence on the outcome that is received on trial n+1 (which is not the case; only the action on trial n+1 determines the outcome of trial n+1). A positive GRS effect indicates that, in line with these considerations, reward on trial n can increase the unrelated choice that is made one trial later, on trial n+1. Such positive GRS effects are therefore not optimal for this task. However, it can be beneficial in environments that do have such dependencies across trials. Often natural environments are structured in multi-step action sequences [88] and in such a setting, positive GRS effects might be adaptive.

**Reviewer 2:**

**Based on their results of earlier studies with nonhuman primates, the authors have combined three studies in this manuscript: (1) Adolescents and young adults performed a behavioral task online on reward guided choices (as established in earlier studies by the same authors). (2) Data from the Human Connectome Project was used to investigate grey matter maturation in the lateral prefrontal cortex, the medial prefrontal cortex and the amygdala in the same age group as participants in study 1. (3) Lesion patients performed the same task as participants in study 1.
Overall, each individual study seems well conducted and analyzed. I have to admit that I am not able to judge study 2 in detail because I lack hands-on expertise in the methods employed. 
The combination of the findings into one manuscript is laudable and points to an underappreciated role of the lateral prefrontal cortex in (the age-related development) of what the authors call "local and global reward states" in their decisions to switch between choice options that fluctuate in their reward probabilities.**

*We thank the reviewer for their positive assessment. We appreciate that the reviewer acknowledges our experimental approach and how we combine several modalities to gain insight about the neurodevelopmental mechanisms of reward learning.*

**1.      Links between studies: As written above, I truly appreciate the authors' effort to combine these three studies. Nevertheless, my general comment is that the authors should be a bit more specific in developing their hypotheses and their rationale for combining these studies. In my view, the motivation and the inspiration, which in large parts is based on findings from nonhuman primates, becomes clear throughout the manuscript. But it took me quite a bit to grasp this rationale. Let me list some suggestions for rather simple clarifications.**

*Thanks for highlighting this and for making very concrete suggestions how to improve our manuscript. We have included these comments as described in detail below. In fact, we went beyond the changes suggested and incorporated more far-reaching changes that we think are very much in line with the suggestions of the reviewer. These changes relate back to the work in nonhuman primates that indeed was the inspiration for the work reported here. These reward learning studies in non-human primates examined the effects of lateral prefrontal lesions on the ability to form associations between (local and global) reward and choices. We make the link to the macaque work clear from the start of the manuscript. However, importantly, we also include additional data in intact and lesioned macaque monkeys using the identical analysis pipeline that we used for our main experiment in adolescents. This additional study (1) demonstrates a striking pattern of similarities but also differences in the way humans and macaque monkeys solve the same probabilistic reversal learning task and (2) enable us to make the link to the macaque work suggested by the reviewer even more directly.*

*Note that in parallel to including this additional study in our manuscript, we decided to move the cross-sectional study investigating grey matter maturation to the supplementary material. We did this in response to comments from reviewer #3, but also because we realised that the reviewer did not find this study very informative. The new study 3, which we added to the manuscript after moving the grey matter analysis to the supplements, is included in the results section as follows,* ***Page 16, study 3, Results:***

**Study 3: Macaque lateral frontal lobe lesions change the impact of contingent rewards and the GRS on choice**

Finally, we reanalysed the non-human primate data that has initially contributed to the suggestion that a subregion of the lateral frontal lobe, i.e. the lateral orbitofrontal cortex, is causally important for credit assignment and local reward learning [6,14]. We did this for two reasons: First, to follow-up and confirm an intriguing effect in our data using more closely matched tasks: GRS effects were negative in the human sample reported here, while they were positive in previous macaque work [7]. This meant macaques stayed with a choice more when the GRS was high [7], whereas human participants reported here switched more when the GRS was high. The second reason was to examine, for the first time, whether changes in global reward learning were also apparent after lateral frontal lobe in macaques, like in our human lesion data. We combined our “credit assignment GLM” with several previously published data sets of macaque choice behavior [6,14]. This allowed our analysis to be optimized towards discovering fine-grained effects of the GRS in a uniquely large data set. We used linear mixed effects models (LME) to account for the fact that multiple sessions belonged to the same individual. Overall, we analyzed 190 sessions from intact monkeys, 45 sessions from monkeys with lateral prefrontal lesion, 55 sessions from monkeys with medial prefrontal lesion, from overall 7 monkeys aged 4-10 years. Note, while these lateral lesions targeted orbitofrontal area 11+13 there is strong reason to believe they disconnected lateral area 47/12o and likely other neighboring regions including anterior insula cortex (see [18,59] for discussion, and refer to Methods for specifics of the lesions sites and nomenclature). In other words, just as in our human lesion study, we assume that multiple subregions of lateral frontal lobe that have dissociable functions are affected by the lateral lesions.

**Figure 5. Positive GRS effects in macaques increase after lateral frontal lobe lesions. (A)** In intact monkeys, we found a small, but significantly *positive* effects of the GRS on stay decisions, which was sign-reversed relative to the negative GRS effect we had found in humans. The inset shows the human GRS effect averaged for all ages (11-35 years) in the matched experimental paradigm (see Fig.3, study 1). **(B)** We compared the effects of frontal lobe lesions in the macaque monkey and revealed that the positive GRS effects after lateral frontal lesions were significantly *stronger* compared to medial lesion groups. The pattern mirrored the human lesion results but in the opposite direction, with lateral lesions in humans abolishing the negative GRS effect (inset: GRS effects from Fig.4, study 3; “Lat” abbreviates “Lateral” and “Med” abbreviates “Medial”) (* p<0.05).

Controlling for local reward learning, we found a small, but significantly *positive* effects of the GRS on stay decisions in the baseline data (intercept-estimate=0.007, SE=0.003; χ2(1)=5.122, p=0.024; Fig.5A). Therefore, we indeed found a sign-reversed effect of the GRS in macaques compared to our human participants using matched experimental paradigms and the same “credit assignment GLM”. Moreover, comparing the effects of lateral frontal lobe lesions to medial lesions in macaques, we found that lateral lesions, too, significantly impacted global reward learning. This mirrored the findings from our human lesion study. However, strikingly and in contrast to our human sample, the GRS effects we observed after lateral lesions were significantly *stronger* (rather than weaker) compared to medial lesion groups (estimate-lateral=0.027, SE= 0.009; χ2(1)=5.080,p=0.024; Fig.5B). This finding further strengthens the idea that both species use the GRS qualitatively differently during learning within the context in which these experiments were conducted. While humans rely negatively on the GRS and this capacity is abolished after lateral lesions, positive GRS effects are amplified in macaques after lateral frontal lobe lesions. This supports the contention that the GRS effect reflects a task-adaptive process in humans, that matures during adolescences and is compromised by lesions, whereas in monkeys, the GRS effect leads to a suboptimal ‘spread’ of reward which is even increased by lateral frontal lobe lesions (Fig.6).

**a.      Especially the abstract is unclear. The three studies could be listed.**

*We have simplified the abstract and structured it as suggested by the reviewer. The abstract now reads as follows:*

Reward-guided choice is fundamental for adaptive behaviour and depends on several component processes supported by prefrontal cortex. Here, across three studies, we show that two such component processes, linking reward to specific choices and estimating the global reward state, develop during human adolescence and are linked to the lateral portions of the prefrontal cortex. These processes reflect the assignment of rewards contingently to local choices, or non-contingently, to choices that make up the global reward history. Using matched experimental tasks and analysis platforms we show the influence of both mechanisms increase during adolescence (study 1), and that lesions to lateral frontal cortex (that included and/or disconnected both orbitofrontal and insular cortex) in human adult patients (study 2) and macaque monkeys (study 3) impair both local and global reward learning. Developmental effects were distinguishable from the influence of a decision bias on choice behaviour, known to depend on medial prefrontal cortex. Differences in local and global assignments of reward to choices across adolescence, in the context of delayed GM maturation of the lateral orbitofrontal and anterior insula cortex, may underly changes in adaptive behaviour.

**b.      The introduction could start with a summary of the findings from nonhuman primates.**

*We have now summarised the findings from nonhuman primates in the second paragraph of the introduction and make it clear that the developmental study is motivated explicitly be these animal models in the context of a protracted and uneven maturation of a network of brain regions involved in learning and decision making. As described above, the inclusion of the new macaque study allows us to be much more explicit about the link to the non-human primate literature and allows us to directly relate our results to them. We have fundamentally restructured and rewritten the entire introduction along the lines suggested.* ***Page 4-7, Introduction.***

**Introduction**

A distributed network in the human brain supports learning from reward and making adaptive decisions. This network comprises several regions in lateral and medial prefrontal cortex (PFC), including lateral and medial orbitofrontal/ventromedial prefrontal cortex, anterior cingulate cortex and subcortical regions including the amygdala and insula. In concert, they contribute component parts to adaptive behaviour such as contingency learning, value comparison, and value representations [1–8]. However, so far, we only have rudimentary knowledge about the developmental dynamics of this brain network and accompanying behavioural changes during adolescence and early adulthood [9,10].

Here we focus on the development of component processes of reward learning that have been strongly linked to neighbouring regions of orbitofrontal and anterior insula cortex in studies of non-human primates: Local and global reward learning. Local reward learning refers to the ability to form contingencies between choice options and outcomes, repeating choices that led to positive outcomes and omitting choices that led to negative outcomes [11–13] (also referred to as ‘contingent reward learning’ or ‘contingent credit assignment’). By contrast, global reward learning, refers to a parallel mechanism where reward simultaneously reinforces not only the choice that caused it but also unrelated choices made in close temporal proximity [6,7,14–17]. This non-contingent global reward learning involves forming a representation of the general reward state (GRS), i.e. how much reward was received overall recently independent of the specific choices that caused them [7]. Lesion studies in macaques and human patients have consistently causally linked local reward learning to lateral orbitofrontal cortex [6,7,14,17], and this is even engrained in variations of gray matter volume in these regions [18]. By contrast, global reward learning mechanisms have been associated with BOLD activity in neighbouring anterior insula cortex [7]. Notably, the function of both these regions contrasts with medial orbitofrontal/ventromedial prefrontal cortex which harbours a variety of value signals linked to value comparison and decision-making processes as opposed to learning [14,16,19–25].

Informed by these animal models and the precise functional localisation of these mechanisms, we consider the development of reward-guided learning in the context of the protracted [26,27] [28,29] [30–33] and non-uniform [30,33] structural maturation of the brain. These considerations lead to the hypothesis that specific cognitive abilities, particularly those related to lateral prefrontal cortex, mature later than others, in particular more medial ones [30,33,34]. This temporal mismatch between protracted structural changes in prefrontal cortex and more rapid maturation of subcortical has been suggested to account for increased risk-taking behaviour in adolescence [35,36] and several studies link development of reward-related behaviour to changes in prefrontal-subcortical interactions [37–39]. However, prefrontal cortex has often been treated as a unitary structure, and consequently we only have a coarse understanding of the different speeds at which subregions of the frontal cortex and, in parallel, subcomponents of reward learning mature [40]. With tentative evidence that adolescents differ from adults in terms of local reward learning, for instance in terms of balancing positive and negative feedback [41–43,43–46], it becomes critical to understand the development of reward learning mechanisms in combination with developmental maturation of their neural underpinnings.

Here, we combine behavioural and lesion investigations to suggest an important role for neighbouring subregions of the lateral frontal cortex, specifically orbitofrontal and anterior insular cortex in the development of local and global reward learning. We used the same multi-option probabilistic learning task originally developed in macaques to dissociate local and global reward learning (Fig.1) [4,6,14]. In study 1, we tested a large online sample (overall *n*=422) of adolescents (11-17 years) and young adults (age 18-35 years) and show that both local and global reward learning change during human adolescence. We chose this age range in accord with previous work [47] and in particular in reference to the protracted maturation profile of lateral prefrontal cortex [32]. Our findings suggest that young adults associated choices more strongly with local rewards and were simultaneously negatively influenced by the GRS. The GRS influence became even more negative with age, meaning young adults, more than adolescents, contextualized their choices within the longer-term reward context, and were less likely to persist with a choice if the alternatives afforded by this context were attractive. By contrast, decision bias mechanisms that depend on the ventromedial portions of the orbitofrontal cortex showed no relationship with age.

The finding that the behavioral mechanisms of local and reward learning continue to change during adolescence align well with the non-human primate literature [6,7,14,17] and our knowledge about structural brain maturation in humans: lateral frontal, compared to medial frontal brain regions, appear to continue to mature during adolescence well into adulthood [30,33,34] and hence we would expect functions that depend on this part of the brain to keep changing during this time period as well. However, only manipulation approaches can provide evidence for a causal reliance of a cognitive function on a neural substrate. Study 2 therefore examined the impact of broad lesions to lateral frontal cortex (lesions included and/or disconnected both orbitofrontal and insular cortex) on local and global reward learning. These studies used experimental tasks and analyses pipelines that were tightly matched to study 1 in cohorts of adult patients with medial or lateral frontal lobe lesions. The results indicated that indeed intact lateral frontal cortex is causally important for both local and global reward learning. Finally, in Study 3, we reanalyse non-human primate data [6,14] that has initially suggested that these lateral frontal regions are important for local reward learning, again using matched experimental tasks and analysis pipelines. This uncovered that lateral lesions in macaques (that likely disconnected both orbitofrontal and insular cortex), too, impair global reward learning as well and give new insights into how the GRS guides choices differently in humans and macaques. While humans showed negative GRS effects, macaques showed positive ones. Together, our results suggest that local and global reward learning mature during adolescence (study 1) and that both learning mechanisms causally depend on (subregions within) lateral frontal cortex (study 2,3). This suggests that the protracted neural maturation in lateral frontal regions [30,33,34] is a key driver for the maturation of local and global reward learning during adolescence.

*In addition, to further improve the integration suggested by the reviewer and to link our results more directly to the macaque work, we have included this new conceptual summary figure. It explains the links between our GRS findings across studies,* ***page 19:***

**Figure 6. Conceptual summary of GRS effects across studies. Study 1: Upper two panels.** People pursue a current choice (banana symbol within circle). They also experience the GRS, the global levels of reward afforded by the environment over time (tree symbol on the periphery of the circle). Adolescents switch away from the currently pursued choice if the GRS is high (small arrow pointing outwards). Adults do this contrast effect of the GRS even more strongly. They contextualise the current choice within the set of alternative options and the knowledge that rich alternatives exist makes adults switch away from their currently pursued course of action. This means that the GRS exerts a ‘contrast effect’: current choices appear less valuable if the GRS is very high. The increased reliance on the GRS coincides with grey matter maturation in lateral frontal lobe regions including the anterior insula and lateral orbitofrontal cortex. **Study 2:** **Lower left panel:** Lesions to lateral frontal lobe (affecting multiple subregions) reduces the GRS effect in human adults. **Study 3:** **Lower right panel:** Macaques also contextualise current choices within the GRS. In contrast to humans, macaques use the GRS fundamentally differently. They show ‘spread of effect’: The GRS positively affects the value of a current choice and this makes macaques stay more with a choice if the GRS is high. Strikingly, lesions to lateral frontal lobe (again affecting multiple subregions) *increase*, rather than decrease, the GRS effect in macaques (thick arrow surrounding small arrow indicates stronger GRS effect after lesion to lateral frontal lobe).

**c.      The words "local and global reward states" are initially quite unclear to readers not familiar with the task. For example, it should be mentioned early on that these words refer to temporal (i.e., trial-by-trial) "adjacency" and not to spatial proximity. Also, "state" does neither refer to "neural states" nor to states as in a Markov Decision Process (as far as I understand the wording used here).**

*Thank you for making us aware of these ambiguities. We now summarise the nonhuman primate research and define the terms local and global reward states clearly within this second introductory paragraph. We have also amended the specific terms identified by the reviewer. It now reads ‘temporal proximity’ and ‘value representations’.* ***Page 4, Introduction:***

Local reward learning refers to the ability to form contingencies between choice options and outcomes, repeating choices that led to positive outcomes and omitting choices that led to negative outcomes [11–13] (also referred to as ‘contingent reward learning’ or ‘contingent credit assignment’). By contrast, global reward learning, refers to a parallel mechanism where reward simultaneously reinforces not only the choice that caused it but also unrelated choices made in close temporal proximity [6,7,14–17]. This non-contingent global reward learning involves forming a representation of the general reward state (GRS), i.e. how much reward was received overall recently independent of the specific choices that caused them [7].

**d.      It is unclear whether the three studies were conducted in parallel or serially.**

**e.      Related to the previous point: It is unclear whether the lesion patients were a "convenience sample" or whether they were selectively recruited for this study (e.g., it could be that they were specifically recruited after the completion of study 2).**

*(d) The developmental behavioural study and the neuroanatomical study were conducted in parallel. The lesion studies were completed afterwards.*

*(e) While the patient lesion data (and the additional monkey lesion sample) were both convenience samples, the present developmental behavioural task was based on the experimental paradigms in these previously published studies. The data from the patient lesion were collected to examine functional specialisation in learning and decision making between medial and lateral orbitofrontal cortex (Noonan et al; 2017). While this was already stated in the main text, we have now made this clearer in the methods. The data from the macaque lesion study come from a series of previously published experiments (Walton et al., 2010; Noonan et al., 2010) which all used the same varying probabilistic reward schedules. This data was re-analysed for the present study to specifically examine the impact of lateral lesions on the influence of the global reward state on choice. This precise mapping of experimental paradigm and analysis technique across species allowed us to directly compare the mechanisms via which human adolescents learn with the way macaques and adult humans learn from reward under normal circumstances and after lesions to subregions of prefrontal cortex. We make all this clearer in the main text and the relevant Methods sections.*

***Study 2, human lesion study, page 14, Results:***

In study 2 we re-analyzed behavioural data in adult patients with lateral (n=4) and medial (n=4) frontal lesions using the same experimental paradigm as study 1 (Fig.S6 [16]) and a matched analysis pipeline. As often the case in patient lesion studies the lesions did not adhere to strict anatomical boundaries. The lateral lesions encompassed regions related to local reward learning in lateral orbitofrontal cortex [6,14,17] as well as more posterior regions in the anterior insula linked to global reward learning [7]. While this was a convenience sample, as the data already existed, the developmental behavioural task was designed to specifically align with these previously published experimental paradigms.

***Study 2, human lesion study, page 34, Methods:***

Data from 8 adults (7 female) with focal lesions involving the medial and lateral frontal lobes were re-analyzed for the purposes of the current study. Patients were originally recruited from the Cognitive Neuroscience Research Registry at McGill University to examine the impact of medial and lateral lesions on the specific influence of the credit assignment mechanism [16]. They were free from neurological or psychiatric disease and not taking any psychoactive medication. For further neuropsychological screening and demographic information see [16]. We analyzed choice data from four patients with lesions to lateral frontal lobe (3 female, mean (and SD) age 60.25 (11.4)) and four patients with lesions to the medial frontal lobe (2 female, mean (and SD) age 61.5 (11.0)). Age was not significantly different between the two groups (t_6_ = -0.16, p = 0.880).

***Study 3, macaque lesion study, page 15, Results:***

Finally, we reanalysed the non-human primate data that has initially contributed to the suggestion that a subregion of the lateral frontal lobe, i.e. the lateral orbitofrontal cortex, is causally important for credit assignment and local reward learning [6,14]. We did this for two reasons: First, to follow-up and confirm an intriguing effect in our data using more closely matched tasks: GRS effects were negative in the human sample reported here, while they were positive in previous macaque work [7]. This meant macaques stayed with a choice more when the GRS was high [7], whereas human participants reported here switched more when the GRS was high. The second reason was to examine, for the first time, whether changes in global reward learning were also apparent after lateral frontal lobe in macaques, like in our human lesion data. We combined our “credit assignment GLM” with several previously published data sets of macaque choice behavior [6,14]. This allowed our analysis to be optimized towards discovering fine-grained effects of the GRS in a uniquely large data set.

***Study 3, macaque lesion study, page 36, Methods:***

Data from six male rhesus macaque monkeys (Macaca mulatta), aged between 4 and 10 years and weighing between 7 and 13.5 kg was reanalysed for the purposes of the current experiment. These data were originally collected and analysed in [6,14] to examine the impact of medial and lateral frontal lobe lesions to the influence of the credit assignment mechanism. Six monkeys originally participated in an experiment reported by [6] in which three animals acted as unoperated controls, whereas the other three received bilateral aspiration lateral orbitofrontal cortex lesions following training and presurgical testing. The three unoperated control monkeys, and one additional monkey who had not participated in Walton et al study then participated in [14] and were tested before and after bilateral aspiration lesions of medial orbitofrontal cortex. All animals were maintained on a 12h light/dark cycle and had 24h ad libitum access to water, apart from when testing. All experiments were conducted in accordance with the United Kingdom Animals Scientific Procedures Act (1986).

**f.      The same point applies to the links between studies 1 and 2. Were the datasets from the Human Connectome Project selected after the behavioral results from study 1 were known?**

*The datasets from the HCP were selected in parallel with the behavioural data and planned to complement each other. We have made that clearer in the* ***Supplementary Methods, page 12,*** *as follows:*

In parallel to Study 1, 125 individuals, aged 11-35 years, were sub-sampled from the Human Connectome Project (HCP) Young Adult (HCPYA, *n*=56) and HCP Developmental (HCPD, *n*=69) databases [13].

*In addition, as described above, we have now moved the study with the Human Connectome data to the supplements. In the legend of this supplementary figure, too, we now clearly describe that this study was conducted in parallel to study 1:*

**Figure S5. Delayed grey matter maturation in lateral orbitofrontal and anterior insula cortex relative to other networked learning and decision-making neural nodes.** Study 1 showed significant changes in local and global reward learning across adolescent development and into early adulthood. Here we investigated the potential underlying neural changes by examining grey matter maturation in prefrontal cortex during the same time window as our behavioural sample, 11-35 years (see Supplementary Methods). We considered regions of interest (ROI) that are related to reward processing. Local and global components of reward learning have both been previously linked to lateral orbitofrontal (lOFC) and anterior insula cortex (Ins) respectively [1,3–5]. Medial orbitofrontal/ventromedial prefrontal cortex (mOFC/vmPFC) is causally linked to value comparison mechanisms [3,6,7], while the dorsal anterior cingulate (dACC) is associated with learning from feedback with BOLD activity in this region correlated with adapting learning rate [8]. Finally amygdala (amy) grey matter density increases with experience in reversal learning-like tasks such as ours [9], lesions to the amygdala affect reversal learning [10] and amygdala signals deviations from precise local reward learning [11,12]. Guided by past NHP work we analyzed structural brain data from an independent data set of 125 individuals from the Human Connectome Project data (HCP developmental and young adult data [13,14]), evenly spread out across our investigated age range. We conducted this study in parallel to study 1. Estimates of individual participants grey matter thickness were extracted from anatomical masks of lateral orbitofrontal cortex and medial orbitofrontal/ventromedial prefrontal cortex [15], dorsal anterior cingulate cortex, amygdala and anterior insula. Developmental trajectories of all five regions were compared using ANCOVA analysis and showed significant differential GM patterns across age (F_4,492_ = 12.35, p<0.001). Follow-up sub analyses compared lateral orbitofrontal cortex separately with the other four areas. Adults and adolescents were also compared directly in independent samples t-tests and Pearson correlational analyses. **(A,B)** Supporting our hypothesis we showed that grey matter in lateral orbitofrontal cortex was significantly lower in young adults compared to adolescence (independent samples t-test, t_123_=6.23, p<0.001; Fig.4A) and correlated negatively with age (Pearson correlation, R=-0.47, p<0.001; Fig.4B), with link functions suggesting this relationship was best fit with a quadratic function (Table S1). **(C,D)** The GM trajectory of the anterior insula, a region in which BOLD activity correlates with the GRS in macaques [1,9] also showed a significant relationship with age (independent samples t-test: t_123_ = -4.34, p < 0.001, Pearson correlation, R = -0.39, p < 0.001). Follow-up tests suggest this relationship was best characterised by a quadratic function (Table S1). Direct comparison between the GM trajectories of lateral orbitofrontal cortex and the anterior insula revealed no significant differences between the GM trajectory of the two regions (F_1,123_ = 0.16, p = 0.694). **(E,F)** The medial orbitofrontal/ventromedial prefrontal cortex also showed continued maturation across the age-range sampled (independent samples t-test, t_123_=3.19, p=0.002; Pearson correlation, R=-0.21, p=0.018; Fig.4C,D) with model fits again characterising this relationship as quadratic (Table S1). However, as the ANCOVA results revealed differential developmental trajectories of GM between lateral and medial orbitofrontal cortex, indexed by a significant age x subregion interaction (F_1,123_=9.896, p=0.002) which suggested medial maturation was significantly less pronounced than lateral regions. **(G,H)** By contrast there was no relationship between age and grey matter in the amygdala, a subcortical region heavily connected with lateral orbitofrontal cortex and intrinsically linked to complementary components of local reward learning [10] (independent samples t-test, t_123_ = -0.79, p = 0.43; Pearson correlation, R = 0.03, p = 0.716; Note this did not improve by using a quadratic instead of a linear link function, see Table S1, which replicates past developmental GM studies [16,17]. Direct comparison between the GM trajectories of lateral orbitofrontal cortex and the amygdala showed, as expected, that developmental GM trajectory was significantly more pronounced in lateral prefrontal cortex compared to the amygdala (significant age x region interaction F_1,123_ = 23.37, p <0.001). **(I,J)** Finally, we examined GM trajectory of the anterior cingulate cortex (focusing on the RCZa or more commonly referred to as dorsal ACC). GM in this region did not vary as a function of age (independent samples t-test: t_123_ = -0.75, p = 0.456, Pearson correlation, R = 0.05, p = 0.549). Direct comparison between the GM trajectories of lateral orbitofrontal cortex and the dACC showed, as expected, that developmental GM trajectory was significantly more pronounced in lateral orbitofrontal cortex (significant age x region interaction (F_1,123_ = 24.80, p <0.001). **(K)** An illustration of the between-subjects interaction of the GM maturation (calculated as mean adolescents minus mean adults) between lateral orbitofrontal cortex, medial orbitofrontal/ventromedial prefrontal cortex, amygdala, dorsal anterior cingulate cortex and anterior insula. This highlights the significantly stronger maturation of grey matter in lateral orbitofrontal cortex and anterior insula compared to the other networked brain regions. This pattern suggests the lateral orbitofrontal and anterior insula cortex undergo the most extensive changes during adolescence, findings in line with a general pattern of maturation across adolescence [18,19]. This suggests that cognitive functions supported by these regions may also undergo more pronounced changes during development compared to those supported by the other areas in the learning and decision-making network. (“x”s indicate individual participants; plots show mean -/+SEM; solid line in the right plots indicate a linear fit. Dashed line represented 95^th^ % confidence interval. * p<0.05, ** p<0.001).

**2.      Analyses and modelling of the behavioral data: The authors conducted a fine-grained series of analyses. In particular, they use three sets of regressors (CxR-history, C-history, and GRS). They also test the choice frequency of the "highest value-option (as defined by value estimates from a Rescorla-Wagner based reinforcement learning model, see Methods)."**

**a.      I may have missed this, but I could not find more details on the Rescorla-Wagner model in the methods.**

*Apologies, the RL model was briefly described in the previous figure legend of Supplementary Figure S2 in the context of the biased by the irrelevant alternatives analysis. Unfortunately, its reference in the main text was mislabelled as still being included in the main methods. We have corrected this reference as well as detailing the model more clearly in the main Methods section of the manuscript* ***Page 31, Methods.***

First level analyses: Reinforcement learning modelling and bias by irrelevant alternatives

In a separate analysis stream, we fitted a reinforcement learning model comprising two model parameters: learning rate and inverse temperature. Specifically, we fit a reinforcement learning model with a Boltzmann action selection rule that uses information about past choices and rewards to estimate expected value for each option on each trial. The reward learning rate and inverse temperature were fitted individually to each session’s data using standard nonlinear minimization procedures. These parameters respectively reflect the weight of influence of the prediction error and the influence of value difference of the probability of choosing an option. To account for outliers in the inverse temperature estimates, we log normalised this parameter after fitting the model. The learning rate and inverse temperature parameters were then submitted to second level analyses.

The reinforcement learning model formed the basis for our investigation of choice biases induced by irrelevant alternatives. This approach investigates whether choices were unduly influenced by the value of an irrelevant alternative. Methodical details are specified in Supplementary Figures S2.

**b.      More importantly, I am wondering if variants of the Rescorla-Wagner model could be directly fitted to the behavioral data. Such models could provide a complementary but more specific - and at the same time more general - outlook on the data than the set of regressors. E.g., the choice of three preceding trials for averaging seems a bit arbitrary; fitting a learning rate as a free parameter could be more generic. Have the authors tested some of these models in the past? Such models would allow to correlate fitted parameters with age.**

*Thank you for this suggestion. We acknowledge that the choice of three preceding trials for averaging in the GRS GLM is a specific choice. However, we have conducted new control analyses to show that the GRS effects exist independent of that specific choice. We did this by varying the history length over which the GRS is calculated. We show that the effects are stable over a wide range of reward histories. This shows that our results do not rely on arbitrary analysis choices. In these control analyses we consider history lengths of 2, 4 and 5. We repeat our main GLM using these lengths and then aggregate the results. We can show that all key results from main GLM replicate in this control analysis:*

1) *Adolescents have a negative GRS effect (t_153_ = -2.79, p = 0.006)*
2) *Adults, too, have a negative GRS effect (t_176_ = -5.27, p < 0.001)*
3) *Importantly, as in our main analysis, adults have a more negative GRS effect than adolescents (F(1,331) = 8.14, p = 0.005; main effect of age group in 2 [age group: adolescents, adults] x 3 [reward history length: 2,4 or 5] repeated measures ANOVA – interaction and main effect of reward history length are not significant)*
4) *Again, as in our main analysis, this developmental trajectory also manifests in a negative correlation between age and GRS effect (r = -0.19, p < 0.001)*

*Importantly, these analyses all use a history length that is different from the one in the main GLM. However, if anything our effects of interest get stronger rather than weaker. This strongly demonstrates that our results do not depend on arbitrary modelling choices. We have now included these new results and details about how we ran this analysis in a new supplementary figure:*

**Figure S4. The effect of GRS on choice is stable across a broad window of reward history length and does not depend on arbitrary statistical choices**. In our main GLM, the GRS is calculated as the arithmetic mean of rewards occurring during the last three trials (see Methods). This history length was chosen a priori based on previous work and based on the number of trials in the experimental schedule. To show that the GRS effects are stable, we repeated our main GLM and varied the length of this reward history between including only the last two trials to including the past five trials; explicitly in these control analyses we consider history lengths of 2, 4 and 5. In accordance with varying reward history length, we adjusted the timescale of the other relevant learning mechanisms (CxR-history and C-history) in the GLM. This ensures that the GRS, CxR-history, and C-history are all calculated over the same set of past trials. Consequently, in the analysis, variance associated with one learning mechanisms is unlikely to be misattributed to another learning mechanism as they cover the same duration of the trial history. For example, when extending the history length of the GRS to five trials, we also extended the history length of CxR-history and C-history by two trials. We then aggregated these alternative regression models and showed that our effects of interest remained significant. Aggregating the results across the 3 alternative choice history lengths we compare the beta weights against zero for adolescents and adults separately in two one-sample t-tests and show negative GRS effects in adolescents (t_153_ = -2.79, p = 0.006) and adults (t_176_ = -5.27, p < 0.001). Importantly, as in our main analysis, adults have a more negative GRS effect than adolescents (F_1,331_ = 8.14, p = 0.005; main effect of age group in 2 [age group: adolescents, adults] x 3 [reward history length: 2,4 or 5] repeated measures ANOVA). Main effects of history length or the interaction between history length and age group were not significant (F_2,666_ = 0.578, p = 0.480, Interaction F_2,666_ = 0.711, p = 0.426). Finally, again, as in our main analysis, this developmental trajectory also manifests in a negative correlation between age and GRS effect (r = -0.19, p < 0.001). For this correlation, we averaged the GRS beta weights across the three GLMS with history length 2, 4 and 5. Critically, these analyses all use a history length that is different from the one in the main GLM and demonstrate that our results do not depend on arbitrary modelling choices.

*This control analysis ensures that our GRS results are independent of specific parameter choices of the GLM. In general, we have found in the past that the GRS results from the GLM are highly consistent with results from a reinforcement learning model that includes parameters capturing GRS effects (Wittmann et al., 2020, Nat Commun). We have not analysed the current data sets with such a reinforcement learning model. However, we do highlight the usefulness doing so in the Discussion and highlight this as a potential future avenue for this research. Moreover, we have clarified the relationship between our results and the simple reinforcement learning model that the reviewer referred to above. The learning rate from the reinforcement learning model is conceptually similar to our local reward learning weights from the regression – both measure the impact an outcome has on the value of repeating the same action and picking the same option again. By contrast, the learning rate is not conceptually related to the GRS effect, which indicates how much people contextualise an outcome based on a longer term average reward rate. We tested these conceptual relationships empirically in our data set. To do so, we calculated a partial correlation between learning rate and local reward learning controlling for age. Indeed, as hypothesized, there was a positive relationship between the residual variance in learning rate and local reward learning (r = 0.35, p < 0.001). By contrast, there was no significant correlation between the learning rate and the GRS effect (r = -0.05, p = 0.340). We clarify the relationships between computational modelling and our GLM in the manuscript now as follows****. Results, study 1, page 14****:*

Note that the effect of local reward learning/contingent reward learning in our GLM is conceptually similar to the learning rate fitted with a reinforcement learning algorithm. Both denote the weight that a new outcome has for updating the value of the corresponding choice [7,55,56]. Higher learning rates, just as a higher local reward learning effect sizes, indicate that an outcome changes the future value of a choice more strongly. Correspondingly, there is a strong positive relationship between the learning rate fitted from our reinforcement learning model and local reward learning (r = 0.35, p < 0.001; correlation of learning rate with CxR_t_). By contrast, the GRS effect is conceptually different from a reward learning rate, because it indicates the effect of a longer-term average reward that is not specifically linked to a choice. Hence, reinforcement learning rate and GRS effect size are uncorrelated (r = -0.05, p = 0.340). The GRS effect hence indicates a qualitatively different effect. Also as expected, neither local nor global reward learning is associated with the inverse temperature from the reinforcement learning model, as the latter indices decision noise rather than the weighting of reward outcomes (inverse temperature vs CxR_t_: r = 0.07, p = 0.168; inverse temperature vs GRS: r = -0.04, p = 0.494).

***Discussion, page 22/23:***

Our results contribute to the debate about the development of reinforcement learning during development [9,44]. Studies indicate that overall the learning rate, i.e. the speed of updating the value of a choice, increases during adolescence [41–43,43–46]. We find the same in our study 1 (Fig.S2). Moreover, the increase in local reward learning in our “credit assignment GLM” could be interpretated along similar lines – as an increase of the weight that an outcome has on changing an option’s value. The strong positive correlation between the learning rate from our reinforcement learning model and local reward learning effect size is a further indication of this. However, studies have started to examine increases in reward learning rate in more detail. As highlighted above, the precise task context plays a big role in whether increased learning rates are observed and if they are desirable to optimise rewards [42]. Another consideration is that learning from outcomes might differ depending on whether that outcome is positive or negative, although these effects, again, appear context-dependent [43,45,83,84]. Our findings that the GRS exerts an increasingly negative effect during development adds to these ideas and highlight influences on reward learning that go beyond changes in a unitary reward learning rate. GRS effects were unrelated to a simple reward learning rate in previous work [7] and also in our current data set. Instead, they contextualise a current choice based on the global reward environment. This mechanism can add to the changes in value observed for a choice and can, in effect, lead to different effective learning rates for positive and negative outcomes [7,51,52]. The negative GRS effects observed here predict higher learning rates for positive outcomes if the GRS is low, and higher learning rates for negative outcomes if the GRS is high. A promising avenue for future research would be to follow-up on these predictions and conduct a more formal modelling analysis of the developmental GRS effects. It might help explain diverging results by suggesting that analyses of reward learning rates should take into account the global reward levels present in the experiments. Neurally, our results suggest that subregions within lateral frontal cortex, specifically anterior insula and orbitofrontal cortex, are particularly promising target regions to look for neural correlates of the development of reinforcement learning mechanisms. These subregions have been shown to integrate rewards with different time constants in adults [7,60,80] and, in adolescents, higher learning rates to negative prediction errors are linked to greater activity in the anterior insula [43].

**3.      Specificity of the task: I am wondering how much task performance across participants differing in age or lesion site depends on the task itself. In particular, on the volatility or the rate of how fast the reward probabilities change (traces in Figure 1B).**

*We thank the reviewer for raising this point. It is interesting to think about which features of the task are particularly important for probing the individual difference in local and global reward learning that we report. Importantly, the task itself and the reward schedules were identical for the adolescents and adult sample, and very similar to both the human lesion and the macaque work (details below). Within each of the studies we report, we use identical tasks and reward schedules, ensuring that any comparison between age groups/lesion groups reflect individual differences rather than task features. Also, across studies, tasks and reward schedules were very similar and were in fact all developed based on the nonhuman primate experiments. They only differed in small aspects that were important for the implementation of each study (e.g., the online study was short to avoid participant dropout, but the lesion experiments were longer as only few testing sessions were available per patient). Therefore, the key results from our studies cannot be explained as function of differences in the tasks used.*

**a.      How did the authors choose the changes in reward probabilities? Did they vary these in earlier studies?**

*The reward schedule was adapted from one of the above macaque studies included in previous published work (Noonan et al. 2010). It was designed to encourage exploration of the reward environment. Reward probabilities drifted slowly and unpredictably drifting over time and ranged between 0 and .9. The reward schedules used in the human patient study were similarly based on the original macaque reward schedule and also varied probabilistically over the testing session. Because testing sessions were shorter in humans than macaques, and because we were concerned that humans would get fatigued/bored in the task if there were prolonged periods with nothing new to learn, the contingency reversals occurred more quickly (25 trials versus 50 or 150 trials, respectively) and sometimes more frequently (3 vs 1 or 3, respectively) in humans than macaques.*

*While this was stated in the manuscript already, we have now summarised the key points collectively in the following way.* ***Results, study 1, page 7:***

We collected developmental data from human participants on a well-characterised 3-choice probabilistic decision-making task (Fig.1) adapted from paradigms previously used in macaques and adult humans [6,14,16,22,48]. As in past studies, here participants made choices in an environment in which the reward probabilities varied probabilistically, and reward contingencies reversed at specific times in the task.

**b.      It would be interesting to speculate (or even to test) if differences between participants would disappear with lower (or higher) volatility.**

*The reviewer raises a very interesting question. We agree that the GRS effect might vary with the effect of volatility and that the influence of various different mechanisms, including the GRS, may be dependent on environmental reward context. We therefore speculate in the discussion that the GRS may be dynamic according to the structure of the environment. Indeed, in more blocked designs the GRS may be less informative than in quickly changing environments and so we would speculate less effect if the trial order was manipulated in a less systematic way.*

***Discussion, page 22:***

Indeed, the GRS may be dynamic and dependent on the structure of the reward environment. In the current experiment reward schedules across all three studies were probabilistic and variable. In more blocked designs the GRS may be less informative than in quickly changing environments and so be less influential on the current choice.

**c.      This may be outside the scope of this manuscript: Simulations with the help of Rescorla-Wagner models (as suggested in comment 2) could help to see in which task situations particular participants would perform the best/worst. This could give further indications about the role of the lateral prefrontal cortex. Such simulations could also provide clearer links between the Rescorla-Wagner models and the set of regressors.**

*This is an interesting idea and we discuss this idea in the discussion. As highlighted above, the strength of the presented work derives from our multi-pronged approach to understanding the neurodevelopmental mechanisms of reward learning. We use a highly similar experimental task in a developmental sample, human lesion patients, and now also intact and lesioned non-human primates. We analyse these data with an established GLM model (Wittmann et al., 2020, Nat Comms) that allows us to dissociate local and global reward learning. We therefore agree that the comprehensive reinforcement learning modelling as well as simulation analyses are outside of the scope of the current manuscript. However, we do agree that this is a valuable approach and worth pursuing in the future. There are additional advantages of modelling these effects with a reinforcement learning framework, and we agree this could and should be done in the future. We have highlighted these points in the Discussion as shown above on page 21.*

**4.      Anatomical masks: I am wondering about grey matter changes in slightly more dorsal parts of the medial prefrontal cortex. As far as I know, these more dorsal regions also change quite substantially during adolescence - and are involved in decision-making.**

*The reviewer raises an interesting point and to address this we have now expanded the GM regions we examine and compare to the trajectory of the Lateral PFC. We extracted GM maturation indices from the dACC region defined by Neubert and colleagues in the same anatomical parcellation study as the OFC masks. This region corresponds to the anterior rostral cingulate zone (RCZa) and is known to activate in human imaging studies in learning and updating values of choices. This area showed no correlation with age and showed a significantly different trajectory compared to the Lateral PFC.*

*Finally, in response to R3.5b we also now include a mask to examine the GM trajectory of the anterior insula and compare directly with the lateral OFC. As predicted from the behavioural results with the influence of the GRS increasing across age the anterior insula shows a significant relationship with age and no significant difference to the trajectory of the lateral OFC.*

*Please refer to figure S5 above where all this is laid out in detail.*

**Reviewer 3:**

**This study combines behavioral, anatomical, and lesion data from three separate studies to argue that distinct local and global components of reward learning develop over adolescence and into young adulthood, and that these behavioral changes are linked to the maturation of lateral prefrontal cortex (LPFC). The authors first use an established probabilistic reward learning paradigm and modeling methods in a large online behavioral study to show that learning of both local reward contingencies, often referred to as credit assignment, and of the global reward state (i.e., average recent reward history regardless of choice) strengthens across development. They then test an a priori hypothesis based on prior work in humans and non-human primates that these behavioral effects depend specifically on LPFC through separate analyses of gray matter maturation across development and behavior on the same task in human lesion patients. Specifically, they use a subsample of the Human Connectome Project (HCP) dataset to show that LPFC gray matter (GM) volume matures (decreases) throughout the same age range as the large-scale behavioral study (11-35), that that this effect is larger than maturation in medial prefrontal cortex (MPFC), and that an amygdala control region does not change over the same age range. Lastly, they show that patients with lesions in LPFC are impaired in both local and global reward learning compared to those with MPFC lesions. By combining these multiple lines of evidence, the authors argue that LPFC is "a strong potential candidate to underlie the behavioral differences in local and global reward learning" seen across this period in development.**

**The manuscript is well written and addresses the important issue of the development of reward learning and decision making, which is of interest to a wide audience of neuroscientists, psychologists, and developmental researchers. The authors apply a relatively novel framework based on computational modeling of reward learning that separates local learning about specific choices from global learning about the overall reward environment. This distinction has potential utility for understanding the role of risk and uncertainty in adolescent decision making, which justifies exploration of these effects across development. Another strength of the paper is that this framework can be interpreted through the lens of optimal or adaptive foraging behavior, and the authors do a good job of interpreting these two components of learning and their behavioral results from the perspectives of behavioral ecology and evolution, which also helps broadens the potential audience.**

**The most important findings in the study are from the behavioral study, which shows nicely that both local and global reward learning improve from adolescence into young adulthood. These conclusions are robust thanks to multiple converging results, including both group comparisons between adolescents and young adults and linear correlations across both age ranges. Another methodological strength is the use of model comparisons to determine whether linear and quadratic functions explain behavioral and neural changes across time, though it is unclear how to interpret the relationships between linear changes in local and global reward learning and quadratic changes in overall task performance and gray matter maturation. The authors also include control analyses showing age is unrelated to the choice history regressor in the credit assignment GLM, the inverse temperature parameter from standard reinforcement learning models ("decision-making noise"), and the "bias by irrelevant alternative" effect previously linked to MPFC (Noonan et al., 2017).**

*We thank the reviewer for their very thorough and constructive comments. We have approached each one carefully. For readability, we have also numbered the points raised so that it is easier to refer to them.*

**1. Additionally, the correlation between these two components of learning across participants is interesting and suggests their development may be linked, but this link could be mediated by other variables besides LPFC maturation. In particular, there is a significant increase across development in the learning rate parameter from the reinforcement learning model, so it would be interesting to know whether this general parameter mediates either or both of the local or global reward learning effects. If it mediates both effects, this could explain the correlation between the two metrics, and one could ask why researchers should study local and global learning parameters instead of the single, more general (and parsimonious) learning rate. On the other hand, showing that the effect on general learning rate explains only one or neither would demonstrate the additional predictive power of local contingency learning and global reward state variables and emphasize their importance in studies of reward learning and value-based decision-making. Relatedly, the authors could improve the interpretation of these constructs and establish their utility by showing that improvements in local and/or global reward learning predict success on the task (e.g., total rewards, % best choice, etc.), especially since these performance metrics also improve across development (Fig. 2).**

*The reviewer raises an interesting point as to whether and how local and global reward learning interact with each other. This is an under researched area across the field, even in adults. Anatomical functional localisation of these parameters and mechanisms speaks against this idea that both processes rely on an identical neural correlate. Local and global reward processing are associated with BOLD activity in different parts of lateral prefrontal cortex. With that in mind we followed the reviewer’s suggestion and examined the relationship between local and global reward learning and the reinforcement learner’s learning rate. The learning rate from the reinforcement learning model is conceptually similar to our local reward learning weights from the regression – both measure the impact an outcome has on the value of repeating the same action and picking the same option again. By contrast, the learning rate is not conceptually related to the GRS effect, which indicates how much people contextualise an outcome based on a longer-term average reward rate. We tested these conceptual relationships empirically in our data set. To do so, we calculated a partial correlation between learning rate and local reward learning controlling for age. Indeed, as hypothesized, there was a positive relationship between the residual variance in learning rate and local reward learning (r = 0.35, p < 0.001). By contrast, there was no significant correlation between the learning rate and the GRS effect (r = -0.05, p = 0.340). We clarify the relationships between computational modelling and our GLM in the manuscript now as follows****. Results, study 1, page 14****:*

Note that the effect of local reward learning/contingent reward learning in our GLM is conceptually similar to the learning rate fitted with a reinforcement learning algorithm. Both denote the weight that a new outcome has for updating the value of the corresponding choice [7,55,56]. Higher learning rates, just as a higher local reward learning effect sizes, indicate that an outcome changes the future value of a choice more strongly. Correspondingly, there is a strong positive relationship between the learning rate fitted from our reinforcement learning model and local reward learning (r = 0.35, p < 0.001; correlation of learning rate with CxR_t_). By contrast, the GRS effect is conceptually different from a reward learning rate, because it indicates the effect of a longer-term average reward that is not specifically linked to a choice. Hence, reinforcement learning rate and GRS effect size are uncorrelated (r = -0.05, p = 0.340). The GRS effect hence indicates a qualitatively different effect. Also as expected, neither local nor global reward learning is associated with the inverse temperature from the reinforcement learning model, as the latter indices decision noise rather than the weighting of reward outcomes (inverse temperature vs CxR_t_: r = 0.07, p = 0.168; inverse temperature vs GRS: r = -0.04, p = 0.494).

*We thank for the reviewer for this suggestion. We think it adds important insights to our analysis by making the relationship between our “credit assignment GLM” and a standard reinforcement learning model more explicit. In addition, we now show that local and global reward learning mechanisms are associated with task success. Controlling for age there were significant positive correlations between local reward learning and the total rewards earned on task (r = 0.244, p < 0.001) and proportion of best choices (r = 0.543, p < 0.001). This pattern was mirrored for global reward learning with a negative correlation with total rewards earned that trended towards significance (r = -0.10, p = 0.069) and a significant negative relationship with proportion of best choices (r = -0.17, p = 0.001). We now include this in the manuscript* ***Results, study 1, page 14:***

Interestingly, negative GRS effects and positive CxR effects were negatively correlated across participants (Pearson correlation; R=-0.16, p=0.002; Fig.S3) and both mechanisms correlated with broad task success. Independent of age there were significant positive correlations between local reward learning and the total rewards earned on task (r = 0.244, p < 0.001) and proportion of best choices (r = 0.543, p < 0.001). This pattern was mirrored for global reward learning with a negative correlation with total rewards earned that trended towards significance (r = -0.10, p = 0.069) and a significant negative relationship with proportion of best choices (r = -0.17, p = 0.001). This pattern of results indicates that participants who performed particularly well in linking local rewards with the specific choices that caused them, also had more negative GRS effects. This suggests that both aspects of reward learning, local assignments of reward and the ability to switch away from unrewarded choices more easily if the reward environment was rich, constituted complementary aspects of task-adaptive behaviour with both processes significantly and simultaneously gaining more influence over behaviour during adolescence. Importantly, our GRS effects of interest also remain stable when varying the history length over which the GLM is calculated (Fig.S4).

**2. Perhaps the most novel and interesting finding reported in the manuscript is the negative effect of global reward state (GRS) on win-stay lose-switch (WSLS) behavior, which increases across development and was confirmed via both credit assignment GLM and ANOVA-based analyses. This is remarkable given that a positive effect in the opposite direction was previously reported in non-human primates (NHPs; Whittmann et al., 2020). The negative relationship found here is optimal in the current task and, as argued at the end of Study 1 in the Results section, is considered adaptive in the context of behavioral ecology, though this seems to slightly contradict arguments presented in the previous paper by the same first author that the positive effect observed in NHPs was potentially adaptive in natural environments with serial dependencies. Regardless, this species difference is a striking finding that deserves to be highlighted, and it will be interesting to see if it replicates across different task structures and whether this pattern is unique to humans.**

*We agree with the reviewer that the negative GRS effect in humans is particularly interesting. In light of this comment, and also comments below and from reviewer 2, we have changed our manuscript extensively to flesh out this point more. We have made two big changes: (1) We now include a reanalysis of previously published non-human primate data (Walton et al., 2010; Noonan et al., 2010) to confirm the species differences and replicate Wittmann’s original positive GRS effect in these monkeys. (2) We have changed the framing of our manuscript and completely rewritten the introduction to orient the paper more strongly along the lesion studies and the link to the non-human primate work.*

(1) *Reanalysis of previously published non-human primate data*

*This dataset also included macaques with aspiration lesions to medial and lateral orbitofrontal cortex. The lateral orbitofrontal cortex lesions, while originally designed to target area 11+13 are now believed to have additionally disconnected area 47/12o (see Rudebeck et al 2014 and Sallet et al 2020) and likely additional neighbouring areas such as the anterior insula cortex connected via the arcuate fasciculus. This allows us to demonstrate in the current study the causal impact of these lateral lesions on the GRS, showing a loss of the positive GRS effect. In light of the reviewers comments we now reintroduce this study as a complement to the patient lesion data.* ***Page 16, study 3, Results:***

**Study 3: Macaque lateral frontal lobe lesions change the impact of contingent rewards and the GRS on choice**

Finally, we reanalysed the non-human primate data that has initially contributed to the suggestion that a subregion of the lateral frontal lobe, i.e. the lateral orbitofrontal cortex, is causally important for credit assignment and local reward learning [6,14]. We did this for two reasons: First, to follow-up and confirm an intriguing effect in our data using more closely matched tasks: GRS effects were negative in the human sample reported here, while they were positive in previous macaque work [7]. This meant macaques stayed with a choice more when the GRS was high [7], whereas human participants reported here switched more when the GRS was high. The second reason was to examine, for the first time, whether changes in global reward learning were also apparent after lateral frontal lobe in macaques, like in our human lesion data. We combined our “credit assignment GLM” with several previously published data sets of macaque choice behavior [6,14]. This allowed our analysis to be optimized towards discovering fine-grained effects of the GRS in a uniquely large data set. We used linear mixed effects models (LME) to account for the fact that multiple sessions belonged to the same individual. Overall, we analyzed 190 sessions from intact monkeys, 45 sessions from monkeys with lateral prefrontal lesion, 55 sessions from monkeys with medial prefrontal lesion, from overall 7 monkeys aged 4-10 years. Note, while these lateral lesions targeted orbitofrontal area 11+13 there is strong reason to believe they disconnected lateral area 47/12o and likely other neighboring regions including anterior insula cortex (see [18,59] for discussion, and refer to Methods for specifics of the lesions sites and nomenclature). In other words, just as in our human lesion study, we assume that multiple subregions of lateral frontal lobe that have dissociable functions are affected by the lateral lesions.

**Figure 5. Positive GRS effects in macaques increase after lateral frontal lobe lesions. (A)** In intact monkeys, we found a small, but significantly *positive* effects of the GRS on stay decisions, which was sign-reversed relative to the negative GRS effect we had found in humans. The inset shows the human GRS effect averaged for all ages (11-35 years) in the matched experimental paradigm (see Fig.3, study 1). **(B)** We compared the effects of frontal lobe lesions in the macaque monkey and revealed that the positive GRS effects after lateral frontal lesions were significantly *stronger* compared to medial lesion groups. The pattern mirrored the human lesion results but in the opposite direction, with lateral lesions in humans abolishing the negative GRS effect (inset: GRS effects from Fig.4, study 3; “Lat” abbreviates “Lateral” and “Med” abbreviates “Medial”) (* p<0.05).

Controlling for local reward learning, we found a small, but significantly *positive* effects of the GRS on stay decisions in the baseline data (intercept-estimate=0.007, SE=0.003; χ2(1)=5.122, p=0.024; Fig.5A). Therefore, we indeed found a sign-reversed effect of the GRS in macaques compared to our human participants using matched experimental paradigms and the same “credit assignment GLM”. Moreover, comparing the effects of lateral frontal lobe lesions to medial lesions in macaques, we found that lateral lesions, too, significantly impacted global reward learning. This mirrored the findings from our human lesion study. However, strikingly and in contrast to our human sample, the GRS effects we observed after lateral lesions were significantly *stronger* (rather than weaker) compared to medial lesion groups (estimate-lateral=0.027, SE= 0.009; χ2(1)=5.080,p=0.024; Fig.5B). This finding further strengthens the idea that both species use the GRS qualitatively differently during learning within the context in which these experiments were conducted. While humans rely negatively on the GRS and this capacity is abolished after lateral lesions, positive GRS effects are amplified in macaques after lateral frontal lobe lesions. This supports the contention that the GRS effect reflects a task-adaptive process in humans, that matures during adolescences and is compromised by lesions, whereas in monkeys, the GRS effect leads to a suboptimal ‘spread’ of reward which is even increased by lateral frontal lobe lesions (Fig.6).

*In the Discussion we consider the species differences, as well as the impact of task structure on the GRS effect. We are thankful that the reviewer noticed the relationship to our previous work and we are very happy to expand on this point. We have indeed argued that a positive GRS effect can be adaptive in macaques. And we also do think that the negative GRS effect observed in humans are more optimal in the context of this specific task. Here are our thoughts how both findings can be integrated,* ***page 25:***

In all analysed macaque data sets here and consistent with previous work on the GRS [7] and credit assignment [6,17], in macaques, the effect of the GRS on choice was *positive*. This contrasted with the negative GRS effect in human participants (study 1,2). This potential species difference is striking, particularly considering that we used a variant of a widely used probabilistic learning task that was matched across studies. However, species comparisons are inherently difficult to interpret. For example, despite the matched tasks, clear differences persisted in the way subjects were introduced to the study (verbal instructions versus weeks of training) and the setting in which the experiments were conducted. Nevertheless, one interpretation of the observed GRS differences is that human behaviour was more in line with ideas from optimal foraging theory that suggest that a value’s choice should be contrasted with the background reward rate of the environment [49,50,54,60,87]. This can promote optimal choice switching and exploration [51,52,54]. However, this view assumes that participants treated the trials in the experiment as discrete, unrelated instances. In contrast, the positive GRS effect in macaques might suggest that non-human primates do not perceive the task as a series of discrete and unrelated trials. Instead, they might expect inter-trial contingencies, for example, that an action on trial n has an influence on the outcome that is received on trial n+1 (which is not the case; only the action on trial n+1 determines the outcome of trial n+1). A positive GRS effect indicates that, in line with these considerations, reward on trial n can increase the unrelated choice that is made one trial later, on trial n+1. Such positive GRS effects are therefore not optimal for this task. However, it can be beneficial in environments that do have such dependencies across trials. Often natural environments are structured in multi-step action sequences [88] and in such a setting, positive GRS effects might be adaptive.

*(2) Updated framing and introduction*

*We have now summarised the findings from nonhuman primates in the second paragraph of the introduction and make it clear that the developmental study is motivated explicitly be these animal models in the context of a protracted and uneven maturation of a network of brain regions involved in learning and decision making. We have fundamentally restructured and rewritten the entire introduction along the lines suggested.* ***Page 4-7, Introduction.***

**Introduction**

A distributed network in the human brain supports learning from reward and making adaptive decisions. This network comprises several regions in lateral and medial prefrontal cortex (PFC), including lateral and medial orbitofrontal/ventromedial prefrontal cortex, anterior cingulate cortex and subcortical regions including the amygdala and insula. In concert, they contribute component parts to adaptive behaviour such as contingency learning, value comparison, and value representations [1–8]. However, so far, we only have rudimentary knowledge about the developmental dynamics of this brain network and accompanying behavioural changes during adolescence and early adulthood [9,10].

Here we focus on the development of component processes of reward learning that have been strongly linked to neighbouring regions of orbitofrontal and anterior insula cortex in studies of non-human primates: Local and global reward learning. Local reward learning refers to the ability to form contingencies between choice options and outcomes, repeating choices that led to positive outcomes and omitting choices that led to negative outcomes [11–13] (also referred to as ‘contingent reward learning’ or ‘contingent credit assignment’). By contrast, global reward learning, refers to a parallel mechanism where reward simultaneously reinforces not only the choice that caused it but also unrelated choices made in close temporal proximity [6,7,14–17]. This non-contingent global reward learning involves forming a representation of the general reward state (GRS), i.e. how much reward was received overall recently independent of the specific choices that caused them [7]. Lesion studies in macaques and human patients have consistently causally linked local reward learning to lateral orbitofrontal cortex [6,7,14,17], and this is even engrained in variations of gray matter volume in these regions [18]. By contrast, global reward learning mechanisms have been associated with BOLD activity in neighbouring anterior insula cortex [7]. Notably, the function of both these regions contrasts with medial orbitofrontal/ventromedial prefrontal cortex which harbours a variety of value signals linked to value comparison and decision-making processes as opposed to learning [14,16,19–25].

Informed by these animal models and the precise functional localisation of these mechanisms, we consider the development of reward-guided learning in the context of the protracted [26,27] [28,29] [30–33] and non-uniform [30,33] structural maturation of the brain. These considerations lead to the hypothesis that specific cognitive abilities, particularly those related to lateral prefrontal cortex, mature later than others, in particular more medial ones [30,33,34]. This temporal mismatch between protracted structural changes in prefrontal cortex and more rapid maturation of subcortical has been suggested to account for increased risk-taking behaviour in adolescence [35,36] and several studies link development of reward-related behaviour to changes in prefrontal-subcortical interactions [37–39]. However, prefrontal cortex has often been treated as a unitary structure, and consequently we only have a coarse understanding of the different speeds at which subregions of the frontal cortex and, in parallel, subcomponents of reward learning mature [40]. With tentative evidence that adolescents differ from adults in terms of local reward learning, for instance in terms of balancing positive and negative feedback [41–43,43–46], it becomes critical to understand the development of reward learning mechanisms in combination with developmental maturation of their neural underpinnings.

Here, we combine behavioural and lesion investigations to suggest an important role for neighbouring subregions of the lateral frontal cortex, specifically orbitofrontal and anterior insular cortex in the development of local and global reward learning. We used the same multi-option probabilistic learning task originally developed in macaques to dissociate local and global reward learning (Fig.1) [4,6,14]. In study 1, we tested a large online sample (overall *n*=422) of adolescents (11-17 years) and young adults (age 18-35 years) and show that both local and global reward learning change during human adolescence. We chose this age range in accord with previous work [47] and in particular in reference to the protracted maturation profile of lateral prefrontal cortex [32]. Our findings suggest that young adults associated choices more strongly with local rewards and were simultaneously negatively influenced by the GRS. The GRS influence became even more negative with age, meaning young adults, more than adolescents, contextualized their choices within the longer-term reward context, and were less likely to persist with a choice if the alternatives afforded by this context were attractive. By contrast, decision bias mechanisms that depend on the ventromedial portions of the orbitofrontal cortex showed no relationship with age.

The finding that the behavioral mechanisms of local and reward learning continue to change during adolescence align well with the non-human primate literature [6,7,14,17] and our knowledge about structural brain maturation in humans: lateral frontal, compared to medial frontal brain regions, appear to continue to mature during adolescence well into adulthood [30,33,34] and hence we would expect functions that depend on this part of the brain to keep changing during this time period as well. However, only manipulation approaches can provide evidence for a causal reliance of a cognitive function on a neural substrate. Study 2 therefore examined the impact of broad lesions to lateral frontal cortex (lesions included and/or disconnected both orbitofrontal and insular cortex) on local and global reward learning. These studies used experimental tasks and analyses pipelines that were tightly matched to study 1 in cohorts of adult patients with medial or lateral frontal lobe lesions. The results indicated that indeed intact lateral frontal cortex is causally important for both local and global reward learning. Finally, in Study 3, we reanalyse non-human primate data [6,14] that has initially suggested that these lateral frontal regions are important for local reward learning, again using matched experimental tasks and analysis pipelines. This uncovered that lateral lesions in macaques (that likely disconnected both orbitofrontal and insular cortex), too, impair global reward learning as well and give new insights into how the GRS guides choices differently in humans and macaques. While humans showed negative GRS effects, macaques showed positive ones. Together, our results suggest that local and global reward learning mature during adolescence (study 1) and that both learning mechanisms causally depend on (subregions within) lateral frontal cortex (study 2,3). This suggests that the protracted neural maturation in lateral frontal regions [30,33,34] is a key driver for the maturation of local and global reward learning during adolescence.

**3. That said, the discussion of how the conceptual framework and empirical findings help us understand decision making under risk and uncertainty during adolescence is not particularly compelling, and given the emphasis on this topic in the abstract, these arguments could be better developed to integrate the behavioral ecology and developmental perspectives.**

*We appreciate the reviewers encouragement to expand and develop our discussion in this area. To expanded the discussion on the development of local and global reward learning and we link it to changes seen elsewhere in cognitive and behavioural flexibility:*

***Page 22:***

Our results contribute to the debate about the development of reinforcement learning during development [9,44]. Studies indicate that overall the learning rate, i.e. the speed of updating the value of a choice, increases during adolescence [41–43,43–46]. We find the same in our study 1 (Fig.S2). Moreover, the increase in local reward learning in our “credit assignment GLM” could be interpretated along similar lines – as an increase of the weight that an outcome has on changing an option’s value. The strong positive correlation between the learning rate from our reinforcement learning model and local reward learning effect size is a further indication of this. However, studies have started to examine increases in reward learning rate in more detail. As highlighted above, the precise task context plays a big role in whether increased learning rates are observed and if they are desirable to optimise rewards [42]. Another consideration is that learning from outcomes might differ depending on whether that outcome is positive or negative, although these effects, again, appear context-dependent [43,45,83,84]. Our findings that the GRS exerts an increasingly negative effect during development adds to these ideas and highlight influences on reward learning that go beyond changes in a unitary reward learning rate. GRS effects were unrelated to a simple reward learning rate in previous work [7] and also in our current data set. Instead, they contextualise a current choice based on the global reward environment. This mechanism can add to the changes in value observed for a choice and can, in effect, lead to different effective learning rates for positive and negative outcomes [7,51,52]. The negative GRS effects observed here predict higher learning rates for positive outcomes if the GRS is low, and higher learning rates for negative outcomes if the GRS is high. A promising avenue for future research would be to follow-up on these predictions and conduct a more formal modelling analysis of the developmental GRS effects. It might help explain diverging results by suggesting that analyses of reward learning rates should take into account the global reward levels present in the experiments. Neurally, our results suggest that subregions within lateral frontal cortex, specifically anterior insula and orbitofrontal cortex, are particularly promising target regions to look for neural correlates of the development of reinforcement learning mechanisms. These subregions have been shown to integrate rewards with different time constants in adults [7,60,80] and, in adolescents, higher learning rates to negative prediction errors are linked to greater activity in the anterior insula [43].

Our hypothesis that local and global reward learning would increase during adolescence was very much guided by studies of human brain maturation suggesting that lateral parts of prefrontal cortex mature later than medial ones [30,31,33]. Indeed, a selective analysis of complementary HCP imaging data indicated that lateral area 47/12o, as well as the anterior insula, showed a longer developmental maturation profile compared the medial orbitofrontal/ventromedial prefrontal cortex, anterior cingulate cortex and amygdala, showing a significant decrease in grey matter across adolescence that continues well into young adulthood (Fig.S5). Lateral orbitofrontal cortex and the anterior insula cortex are strong potential candidates to underlie the behavioural differences in local and global reward learning seen across the same period of development. While this conjecture is indirect, it is well known that localised regional GM volume correlates with motor, cognitive and social skills [18,27,85,86]. Indeed, we recently demonstrated in macaques that GM around the principle sulcus is causally altered by extended training in discrimination reversal learning, with GM variation in this region related to individual variation in training speed [18]. Longitudinal studies examining grey matter maturation and the development of reward learning in the same sample are needed to provide more direct evidence about this link between cognitive and neural development.

However, even longitudinal studies are usually correlative and as such can only provide limited evidence about causal relationships. Study 2 and 3 therefore used finely matched experimental paradigms and analysis techniques to directly assess the causal importance of lateral frontal regions in local and global reward learning. In humans we show that the GRS effect is reduced (i.e. closer to zero) after lateral prefrontal lesions, compared to medial orbitofrontal lesions, in combination with decreased local reward learning (Fig.4). Note that the direction of change in both local and global reward learning after lesions is highly compatible with the correlation between those two variables in our online sample (Fig.S3). This suggests that both learning processes are at least partly supported by neural mechanisms in lateral frontal cortex. In study 3, we confirm in macaque monkeys that the GRS effect is altered after lateral lesions (Fig.5). The macaque lesion effects, however, were qualitatively different. First, as we know from the same data in past work [6,14], local reward learning is reduced after lateral lesions. This means that both humans and macaques show a decrease of contingent/local reward learning after broad lesions to lateral prefrontal cortex. However, rather than decreasing the GRS effect as in humans, lateral lesions *increased* the GRS effect in macaques. While a negative GRS effect was abolished in humans, a positive GRS effect was increased in macaques (Fig.6). Together with the decline of local reward learning in both species, this leads to the contention that the GRS effect reflects a task-adaptive process in humans, that matures during adolescences and is compromised by lesions, whereas in monkeys, the GRS effect reflects a suboptimal ‘spread’ of reward which is even increased by lateral frontal lobe lesions.

*Page 26:*

Lateral prefrontal regions, beyond orbitofrontal cortex, are also all late to mature across adolescence [30,89]. The more dorsolateral prefrontal regions are associated with intelligence, fluid cognition, working memory and attentional control [90–92]. A critical direction for future work will be to examine the interactions between these developing cognitions, and brain network dynamics, and the learning mechanisms described here. Recent advances in network neuroscience offer exciting methods to characterise individual differences in complex cognitions as a function of local and global brain network topology and community structure [93–95]. Characterising these interactions could ultimately improve predictions of transdiagnostic features of neurodevelopmental and behavioural trajectories.

**4. In contrast, the evidence linking local and global reward learning to LPFC in this manuscript is flawed and provides little confidence that developmental changes in local and global reward learning are related to maturation of LPFC. The biggest issues are related to the weak logic behind the GM volume analyses. Showing that LPFC changes in GM volume in a separate cross-sectional sample from the HCP merely confirms what has been previously published, mainly that maturation of PFC as a whole (e.g., as shown in Gogtay et al., 2004 or Raznahan et al., 2010) also applies to specific LPFC subregions 47/12o and 14. More importantly, showing that two variables (behavior and LPFC GM) change across the same age range in two non-overlapping samples does not provide evidence that those effects are related. Similarly, showing that LPFC is maturing to a greater degree than MPFC over the same period is not sufficient to claim one is more closely related to a behavioral change. Without stronger evidence (e.g., by measuring these trends in the same sample), the claims based on the HCP data are weakly supported.**

*We acknowledge the reviewer’s concerns. We realised that reviewer 2 had a similar criticism. Given the collective response to the HCP results we have taken the decision to move this analysis into the supplementary material to de-emphasise its importance to our logic. As described in the last point, we have now fundamentally rearranged our manuscript and organise it around the parts that have been described as strengths by the reviewer. In the main text, we only briefly refer to the HCP data and, as the reviewer suggests below, we place the bulk of the argument on the relevant citations.* ***Page 15, study 2, Results:***

**Study 2: Local and global reward learning are impaired by lesions to lateral frontal lobe**

The finding that the behavioral mechanisms of local and reward learning continue to change during adolescence align well with the non-human primate literature [6,7,14,17] and our knowledge about structural brain maturation in humans: lateral, compared to medial frontal brain regions, appear to continue to mature during adolescence well into adulthood [30,33,34] and hence we would expect functions that depend on this part of the brain to keep changing during this time period as well. Our own analysis of Human Connectome data [57,58] in a set of selected reward-sensitive regions of interests (ROIs) confirmed that the greatest age-related differences over our investigated age range existed in lateral and not medial regions of the brain’s reward circuitry (Fig. S5). However, only manipulation approaches can provide evidence for a causal reliance of a cognitive function on a neural substrate. Study 2 therefore examined the impact of broad lesions to lateral frontal cortex on local and global reward learning.

*This the figure that we added to the Supplementary material:*

**Figure S5. Delayed grey matter maturation in lateral orbitofrontal and anterior insula cortex relative to other networked learning and decision-making neural nodes.** Study 1 showed significant changes in local and global reward learning across adolescent development and into early adulthood. Here we investigated the potential underlying neural changes by examining grey matter maturation in prefrontal cortex during the same time window as our behavioural sample, 11-35 years (see Supplementary Methods). We considered regions of interest (ROI) that are related to reward processing. Local and global components of reward learning have both been previously linked to lateral orbitofrontal (lOFC) and anterior insula cortex (Ins) respectively [1,3–5]. Medial orbitofrontal/ventromedial prefrontal cortex (mOFC/vmPFC) is causally linked to value comparison mechanisms [3,6,7], while the dorsal anterior cingulate (dACC) is associated with learning from feedback with BOLD activity in this region correlated with adapting learning rate [8]. Finally amygdala (amy) grey matter density increases with experience in reversal learning-like tasks such as ours [9], lesions to the amygdala affect reversal learning [10] and amygdala signals deviations from precise local reward learning [11,12]. Guided by past NHP work we analyzed structural brain data from an independent data set of 125 individuals from the Human Connectome Project data (HCP developmental and young adult data [13,14]), evenly spread out across our investigated age range. We conducted this study in parallel to study 1. Estimates of individual participants grey matter thickness were extracted from anatomical masks of lateral orbitofrontal cortex and medial orbitofrontal/ventromedial prefrontal cortex [15], dorsal anterior cingulate cortex, amygdala and anterior insula. Developmental trajectories of all five regions were compared using ANCOVA analysis and showed significant differential GM patterns across age (F_4,492_ = 12.35, p<0.001). Follow-up sub analyses compared lateral orbitofrontal cortex separately with the other four areas. Adults and adolescents were also compared directly in independent samples t-tests and Pearson correlational analyses. **(A,B)** Supporting our hypothesis we showed that grey matter in lateral orbitofrontal cortex was significantly lower in young adults compared to adolescence (independent samples t-test, t_123_=6.23, p<0.001; Fig.4A) and correlated negatively with age (Pearson correlation, R=-0.47, p<0.001; Fig.4B), with link functions suggesting this relationship was best fit with a quadratic function (Table S1). **(C,D)** The GM trajectory of the anterior insula, a region in which BOLD activity correlates with the GRS in macaques [1,9] also showed a significant relationship with age (independent samples t-test: t_123_ = -4.34, p < 0.001, Pearson correlation, R = -0.39, p < 0.001). Follow-up tests suggest this relationship was best characterised by a quadratic function (Table S1). Direct comparison between the GM trajectories of lateral orbitofrontal cortex and the anterior insula revealed no significant differences between the GM trajectory of the two regions (F_1,123_ = 0.16, p = 0.694). **(E,F)** The medial orbitofrontal/ventromedial prefrontal cortex also showed continued maturation across the age-range sampled (independent samples t-test, t_123_=3.19, p=0.002; Pearson correlation, R=-0.21, p=0.018; Fig.4C,D) with model fits again characterising this relationship as quadratic (Table S1). However, as the ANCOVA results revealed differential developmental trajectories of GM between lateral and medial orbitofrontal cortex, indexed by a significant age x subregion interaction (F_1,123_=9.896, p=0.002) which suggested medial maturation was significantly less pronounced than lateral regions. **(G,H)** By contrast there was no relationship between age and grey matter in the amygdala, a subcortical region heavily connected with lateral orbitofrontal cortex and intrinsically linked to complementary components of local reward learning [10] (independent samples t-test, t_123_ = -0.79, p = 0.43; Pearson correlation, R = 0.03, p = 0.716; Note this did not improve by using a quadratic instead of a linear link function, see Table S1, which replicates past developmental GM studies [16,17]. Direct comparison between the GM trajectories of lateral orbitofrontal cortex and the amygdala showed, as expected, that developmental GM trajectory was significantly more pronounced in lateral prefrontal cortex compared to the amygdala (significant age x region interaction F_1,123_ = 23.37, p <0.001). **(I,J)** Finally, we examined GM trajectory of the anterior cingulate cortex (focusing on the RCZa or more commonly referred to as dorsal ACC). GM in this region did not vary as a function of age (independent samples t-test: t_123_ = -0.75, p = 0.456, Pearson correlation, R = 0.05, p = 0.549). Direct comparison between the GM trajectories of lateral orbitofrontal cortex and the dACC showed, as expected, that developmental GM trajectory was significantly more pronounced in lateral orbitofrontal cortex (significant age x region interaction (F_1,123_ = 24.80, p <0.001). **(K)** An illustration of the between-subjects interaction of the GM maturation (calculated as mean adolescents minus mean adults) between lateral orbitofrontal cortex, medial orbitofrontal/ventromedial prefrontal cortex, amygdala, dorsal anterior cingulate cortex and anterior insula. This highlights the significantly stronger maturation of grey matter in lateral orbitofrontal cortex and anterior insula compared to the other networked brain regions. This pattern suggests the lateral orbitofrontal and anterior insula cortex undergo the most extensive changes during adolescence, findings in line with a general pattern of maturation across adolescence [18,19]. This suggests that cognitive functions supported by these regions may also undergo more pronounced changes during development compared to those supported by the other areas in the learning and decision-making network. (“x”s indicate individual participants; plots show mean -/+SEM; solid line in the right plots indicate a linear fit. Dashed line represented 95^th^ % confidence interval. * p<0.05, ** p<0.001).

*In the Discussion we consider that the developmental behavioural effects in these two mechanisms are likely to depend on the continued maturation of these regions as seen in the parallel GM analysis but that this can only be established in a sample in the same subjects and so thus lays the hypothesis for future studies to test directly in the same sample,* ***page 23:***

Our hypothesis that local and global reward learning would increase during adolescence was very much guided by studies of human brain maturation suggesting that lateral parts of prefrontal cortex mature later than medial ones [30,31,33]. Indeed, a selective analysis of complementary HCP imaging data indicated that lateral area 47/12o, as well as the anterior insula, showed a longer developmental maturation profile compared the medial orbitofrontal/ventromedial prefrontal cortex, anterior cingulate cortex and amygdala, showing a significant decrease in grey matter across adolescence that continues well into young adulthood (Fig.S5). Lateral orbitofrontal cortex and the anterior insula cortex are strong potential candidates to underlie the behavioural differences in local and global reward learning seen across the same period of development. While this conjecture is indirect, it is well known that localised regional GM volume correlates with motor, cognitive and social skills [18,27,85,86]. Indeed, we recently demonstrated in macaques that GM around the principle sulcus is causally altered by extended training in discrimination reversal learning, with GM variation in this region related to individual variation in training speed [18]. Longitudinal studies examining grey matter maturation and the development of reward learning in the same sample are needed to provide more direct evidence about this link between cognitive and neural development.

**5. A second major concern is the use of the broad terms "LPFC" and "MPFC", which are problematic for several reasons. First and most generally, it is important to be as precise as possible with neuroanatomical terminology. As noted several times in the manuscript, two recent NHP papers published by the first author used similar tasks and analyses in two recent NHP papers to show local contingency learning is specific and causally related to area 47/12o in the ventrolateral PFC, and that this effect is dissociable from GRS signals localized to the nearby but distinct anterior insular cortex (AIC) (Whittmann et al., 2020; Folloni et al., 2021). Therefore, it's unclear why the manuscript uses the general term LPFC when the strong evidence for their a priori hypotheses is much more specific. In particular, this deceives the reader into thinking both functions rely on the same region, when in fact these two different cognitive processes have been carefully dissociated in prior studies. For example, the lack of any ROI covering the AIC region linked to GRS in their GM analyses leads the reader to assume that both functions have been localized to the 47/12o ROI. Moreover, the AIC GRS region is not even a subregion of PFC, meaning the broad LPFC label is not even an accurate superset of the two subregions. This framing is therefore oversimplified to the point that it is a disservice to the precision of the prior work and muddies the waters for the field in general, especially for readers not familiar with these prior studies, these local and global components of reward learning, or the relevant neuroanatomy. Similarly, the authors seem to mean a specific ventromedial PFC region when they refer to MPFC (which covers a much larger range of regions and functions), and if that's the case, it would improve the clarity of this manuscript and the literature as a whole to use the more precise term vmPFC instead of MPFC.**

**I suspect the authors made this choice because they wanted to include the human lesion data, which are inherently limited in spatial resolution. These results are valuable and provide causal evidence that local and global reward learning are more specific to lateral than medial regions, which adds an important brain-behavior link that was missing from the gray matter findings. While the diffuse nature of the lesions likely renders this analysis unable to separate the two subregions and dissociate the two cognitive functions, this is reasonable and a standard challenge for human lesion data. The authors could simply acknowledge that these two processes rely on different subregions that are both covered by the LPFC lesions, which would maintain the distinction in the literature and improve the interpretability of the current study. In that case, showing a difference in both behaviors between groups with medial and lateral lesions is still a strong finding. However, another issue is that this difference is hard to interpret without behavioral data from age-matched controls, especially since these lesion patients are older adults that may behave differently than the adolescents and young adults. At the very least, the age of the lesion patients should be included in the Methods section. Additionally, it is generally important to be cautious when interpreting human lesion data because they typically cover multiple subregions as well as neighboring white matter tracts, particularly since these issues and related differences between lesion methods and locations across rodent, NHP, and human data have generated conflicting findings and confusion in the literature (see Sallet et al., 2020 and related reversal learning literature).**

*Thank you for highlighting these important points. We certainly agree that it is important to be as precise as possible with neuroanatomical terminology. And precisely as the reviewer says, when using lesion approaches as we do, the most precise terminology possible is necessarily a very crude one, because the lesions span broad swaths of cortex and white matter. We appreciate the reviewer's suggestions on these points and have addressed them in four ways.*

*Firstly, as we have now moved the grey matter investigations to the supplementary materials, this already solves much of the anatomical ambiguities. The grey matter work has a very precise anatomical resolution on the level of subregions, while the lesion work encompassed damage to wider parts of the brain. This tension is now gone.*

*Secondly, we have now been much clearer in our anatomical labelling to reflect our belief from the start that the GRS depends on the anterior insula cortex and the local reward learning mechanism depends on lateral regions of the OFC. See also the revised introductory paragraphs above that orient the paper more closely based on the anatomical considerations highlighted by the reviewer. We are now explicit in the extents of the patient lesions and the likelihood that they impact not only the lateral regions of the OFC but also the adjacent anterior insula, if not directly through grey matter loss, indirectly through disconnection of the arcuate fasciculus. Furthermore, we now also include macaque lesion data described in study 3. Here we also have good reason to believe the lateral OFC lesions have disconnected neighbouring areas of cortex including the anterior insula and we describe these lesions, the reasoning behind this logic (see Rudebeck et al 2014 and Sallet et al 2020) and the consequences for our interpretation of the anatomical foci of the GRS effects. In the labelling of the two sets of lesions themselves we use the broad terms “Lateral Frontal cortex and Medial Frontal cortex” because the lesions were broad and impacted multiple subregions. We think it is important to remain transparent about the scope of the lesions and this particular limitation. With the data we have, and the nature of the lesions themselves, we cannot distinguish between the GRS effects arising from the damage/disconnection of the anterior insula vs the damage to the lateral orbitofrontal cortex. However, we do agree with the reviewer and also believe there is enough evidence in the literature to strongly speculate about the GRS and contingent reward learning being linked to separate subregions.*

*Here are some examples of changes and clarifications to the manuscript in line with the reviewers requests:*

***Abstract, page 2 (italics highlight relevant parts):***

Using matched experimental tasks and analysis platforms we show the influence of both mechanisms increase during adolescence (study 1), and that lesions to *lateral frontal cortex (that included and/or disconnected both orbitofrontal and insular cortex)* in human adult patients (study 2) and macaque monkeys (study 3) impair both local and global reward learning.

***Introduction, page 4 (italics highlight relevant parts):***

Lesion studies in macaques and human patients have consistently causally *linked local reward learning to lateral orbitofrontal cortex* [6,7,14,17], and this is even engrained in variations of gray matter volume in these regions [18]. By contrast, *global reward learning mechanisms have been associated with BOLD activity in neighbouring anterior insula cortex* [7].

***Introduction, page 6 (italics highlight relevant parts):***

Study 2 therefore examined the impact of broad lesions to lateral prefrontal cortex (*lesions included and/or disconnected both orbitofrontal and insular cortex*) on local and global reward learning.

***Results, study 2, page 14 (italics highlight relevant parts):***

In study 2 we re-analyzed behavioural data in adult patients with lateral (n=4) and medial (n=4) frontal lesions using the same experimental paradigm as study 1 (Fig.S6 [16]) and a matched analysis pipeline. *As often the case in patient lesion studies the lesions did not adhere to strict anatomical boundaries. The lateral lesions encompassed regions related to local reward learning in lateral orbitofrontal cortex [6,14,17] as well as more posterior regions in the anterior insula linked to global reward learning [7].*

*Thirdly, we now include an anterior insular cortex (AIC) mask in our grey matter analysis. This will make clearer that there are multiple subregions in lateral prefrontal cortex. We have moved these analyses to the supplementary material (see above response). There, we now describe neurodevelopmental differences in a more complete network of brain regions associated with learning and decision-making including lateral OFC (area 47/12o), medial OFC (area 14), dorsal ACC (area RCZa), amygdala and anterior insula. We show grey matter in the anterior insula follows a similar delayed developmental trajectory relative to the lateral OFC, again supporting our hypothesis that this region corresponds to the neural substrate of the GRS effects and could be related to the delayed influence of this mechanism.* ***Supplementary Figure S5***

*Forth, we now refer to the medial orbitofrontal area as “medial orbitofrontal/ventromedial prefrontal cortex”.*

*Finally, as requested, we now include the age of the lesion patients in the methods section. Patient groups were age-matched with no significant difference between them (p = 0.880).* ***Page 34, Methods, study 2:***

We analyzed choice data from four patients with lesions to lateral frontal lobe (3 female, mean (and SD) age 60.25 (11.4) years) and four patients with lesions to the medial frontal lobe (2 female, mean (and SD) age 61.5 (11.0) years).

**6. In summary, the manuscript has an interesting and novel set of findings about how local and global components of reward learning change across development, and the lesion data provide evidence that these functions are causally related to lateral regions and not MPFC in humans. However, this brain-behavior link is based on older adults, which leaves the behavioral study as the only convincing developmental findings. While the neuroanatomical language can potentially be improved, the gray matter analyses meant to address brain development are flawed enough that they detract more than they add to the paper. In my opinion, removing the manuscript would be simplified by replacing that analysis with a few strong references. Unfortunately, this leaves the claims linking behavioral changes across development to neural maturation poorly supported. Thus, the manuscript as currently written contains important and novel contributions to human reward neuroscience based on the interesting behavioral effects and lesion data, but with only weak evidence linking these behavioral changes to brain development, it offers fairly limited contributions related to adolescence that would appeal to the wider audience of developmental researchers. Therefore, in my opinion, further evidence is needed to justify framing the manuscript around development, or lacking such evidence, it should be rewritten as a basic reward neuroscience paper.**

*We thank the reviewer for this suggestion. We have in response put more emphasis on the basic neuroscience components of our work. The addition of macaque lesion data enables us to emphasise the basic neuroscience more strongly, too. However, in light of other comments that asked that discussion of the findings was integrated more widely with the developmental literature we felt it was important to maintain a balance between the developmental and the basic neuroscience aspects of our work. We hope it became clear that many of the changes highlighted above were very much driven by this comment of the reviewer. We have (1) fundamentally rewritten our manuscript, (2) moved the contentions study to the supplements and (3) replaced it with a new study that enables direct comparison between human and macaque behaviour on the same task. We thank the reviewer again for their careful reading of our manuscript. We genuinely believe that the suggestions have improved our paper. We have also included the following illustrative figure to illustrate the conceptual relationships of the identified GRS effects across studies.* ***Figure 6, page 18:***

**Figure 6. Conceptual summary of GRS effects across studies. Study 1: Upper two panels.** People pursue a current choice (banana symbol within circle). They also experience the GRS, the global levels of reward afforded by the environment over time (tree symbol on the periphery of the circle). Adolescents switch away from the currently pursued choice if the GRS is high (small arrow pointing outwards). Adults do this contrast effect of the GRS even more strongly. They contextualise the current choice within the set of alternative options and the knowledge that rich alternatives exist makes adults switch away from their currently pursued course of action. This means that the GRS exerts a ‘contrast effect’: current choices appear less valuable if the GRS is very high. The increased reliance on the GRS coincides with grey matter maturation in lateral frontal lobe regions including the anterior insula and lateral orbitofrontal cortex. **Study 2:** **Lower left panel:** Lesions to lateral frontal lobe (affecting multiple subregions) reduces the GRS effect in human adults. **Study 3:** **Lower right panel:** Macaques also contextualise current choices within the GRS. In contrast to humans, macaques use the GRS fundamentally differently. They show ‘spread of effect’: The GRS positively affects the value of a current choice and this makes macaques stay more with a choice if the GRS is high. Strikingly, lesions to lateral frontal lobe (again affecting multiple subregions) *increase*, rather than decrease, the GRS effect in macaques (thick arrow surrounding small arrow indicates stronger GRS effect after lesion to lateral frontal lobe).

**8. Minor Grammatical points:
Introduction paragraph 2, sentence 3: "particularly in LPFC" should likely be "particularly those related to LPFC" since cognitive ablities cannot be "in" LPFC
Supp. Table 1 legend: "a likelihood ratio tests" grammar
Discussion: "behaviroual " misspelled
Methods: missing verb: "Young adults were additionally via local university online advertisement and via email lists. "; add "to" to "These precise regions have specifically and repeatedly been linked learning and decision-making mechanisms in the adult population "**

*Thank you. These have been amended and we appreciate the reviewer highlighting them.*

**Editors comments
Upon looking over the other reviewer comments, I have a few additional thoughts:
*       The behavioral analysis approach, including the conceptual framework and findings, seem likely to be useful in the debate over decision making in adolescence, so if the concerns around that study and its interpretation are addressed, this is already a strong behavioral paper. Adding the lesion data to incorporate the "biology" parts makes it a very nice reward neuroscience paper. The gray matter analyses are not convincing to me, but I'm not an expert in that field so perhaps that result is more novel than it seems. I think if the authors did a better job of integrating the results from the three studies, the gray matter result could be positioned as tenuous evidence that the lesion result is in a region that develops in this age range.**

*Thank you for these additional comments. In line with the comments of the three reviewers and your own constructive feedback we have aimed to strengthen the integration of the different studies in each section of the manuscript; abstract, introduction, results and discussion. In particular, we have moved the grey matter study to the supplements. We realised that two reviewers were not convinced by it and thus decided to de-emphasize these results by putting them in the supplementary material*

*       **I do wish there was more discussion of how these findings integrate with the developmental literature, but I also recognize that fully developing arguments around how those issues can be viewed through their conceptual framework is a tall task that would be tough to fit in a reasonable Discussion section and may better fit as a review/position/opinion paper.**

*Thanks for this suggestion. We have taken it onboard. In line with reviewer 3’s comments to restructure the paper as a basic neuroscience paper we have tried to strike a balance between the basic science and the developmental literature. However, we have expanded the discussion on the development of local and global reward learning and we link it to changes seen elsewhere in cognitive and behavioural flexibility. We have copy/pasted the changes we made above in response to reviewer 1 and 3.*
