# Supplementary material for: Local and global reward learning in the lateral frontal cortex show differential development during human adolescence
Source: PLoS Biol. 2023 Mar 2;21(3):e3002010. doi: 10.1371/journal.pbio.3002010 (PMC10013901; doi:10.1371/journal.pbio.3002010)
Supplement: Attachment — Submitted filename: ResponseToReviewers.docx [file pbio.3002010.s004.docx]

Reviewer remarks:

Reviewer's Responses to Questions

Do you want your identity to be public for this peer review?
Reviewer #2: Yes: Christoph Korn
Reviewer #3: Yes: Colin W Hoy

*We are grateful to reviewer 1 and 2 for their support.*

Reviewer #2: All my comments have been addressed.


Reviewer #3: Overall, this is a strong manuscript that makes combined behavioral and neural contributions to the field of reward learning and development. I would like to thank the authors for their efforts to revise and improve the manuscript, which have addressed all of my major concerns. This involved major reorganization of the manuscript, including revisions to the structure of their arguments in the introduction and discussion, as well as new data, analyses, and results. I believe that the additional analyses clarifying the relationship between local and global reward learning and more broadly used reinforcement learning parameters such as learning rate will help contextualize these concepts in the wider field, and the new language helping interpret these findings from the perspectives of behavioral ecology and development ensure those advances are accessible to audience beyond cognitive neuroscience. The HCP GM volume data is appropriately still included as a supplement, and breaking that analysis down according to the specific subregions associated with local and global reward learning respectively improves their contribution. More generally, the authors are careful to use specific neuroanatomical terms when possible but general terms when necessary (i.e., referring to lesion locations). Most importantly, the new non-human primate lesion and behavioral data and analyses reinforce and clarify the striking species differences in the effect of GRS on decision-making, strengthening a major contribution to the field. See below for minor comments and suggestions, but I would recommend this manuscript for publication and would be comfortable with the authors making these minor corrections without further consultation of the reviewers.

*We thank the reviewer for their supportive and constructive comments which we have addressed in turn below and highlight changes in the manuscript in red.*

Minor issues:

1. In the introduction, the authors refer to the insula as a **subcortical region** when it is in fact a cortical region (sentence 2).

*Thank you for highlighting this mistake in the re-write. The sentence has now been amended to the following*

This network comprises several regions in lateral and medial prefrontal cortex (PFC), including lateral and medial orbitofrontal/ventromedial prefrontal cortex, as well as other areas such as anterior cingulate cortex, insula cortex and the amygdala.

2. In the results, Fig. 1a shows **two examples of positive feedback** instead of a positive and negative example. A similar issue appears in Sup. Fig. 7a.

*Thank you for spotting this. Figure 1a has been amended to show correct (upper panel) and incorrect feedback (lower panel). However, Figure S7a is correct. Monkeys were shown three stimuli and regardless of whether they were correct or incorrect the chosen stimulus remain onscreen during the feedback phase. The two events are distinguished by the correct response being noted by an auditory tone and rewarded with a sucrose pellet. Both these events are illustrated in the upper panel.*

3. For Supplemental Fig. 4, it's a **little unclear how the results were aggregated across the analyses using different history lengths** to make the correlation plot, but perhaps I missed that somewhere. If not, that language in the methods or caption could be further clarified.

*Apologies this was not as clear as it could have been. For each subject we averaged the beta weights derived from the analyses with a reward history window of 2 trials, 4 trials and 5 trials. These average GRS beta weights were then plotted and correlated against the subjects’ ages.*

**(D)** Finally, again, as in our main analysis, this developmental trajectory also manifests in a negative correlation between age and GRS effect (r = -0.19, p < 0.001). For this correlation, we averaged the GRS beta weights, within each subject, across the three GLMS with history length 2, 4 and 5. The average GRS beta weights were then plotted against age. Critically, these analyses all use a history length that is different from the one in the main GLM and demonstrate that our results do not depend on arbitrary modelling choices.

Optional suggestions:

1. The language emphasizing the differences between the effect of lateral frontal lesions making the effect of GRS on local reward learning weaker vs. stronger for humans vs. NHPs respectively is appropriate, but there's also a point of potential reconciliation: the overall direction of the effect (more positive GRS effect) is consistent across species. Obviously, the baseline difference changes the interpretation, but is it possible LPFC is playing a general role in contextualizing GRS to avoid the "spread of reward"? **Perhaps this effect is already strong enough in humans to create a negative overall effect, but this function degrades in both species after LPFC lesions**. Granted this perspective is speculative, but I just wanted to bring it to the authors attention as a potential discussion point.

*Thank you for suggesting this potential point of convergence. We have now included the argument in the discussion.*

In study 3, we confirm in macaque monkeys that the GRS effect is altered after lateral lesions (Fig.5). However, the macaque lesion effects appear qualitatively different. First, as we know from the same data in past work [6,14], local reward learning is reduced after lateral lesions. This means that both humans and macaques show a decrease of contingent/local reward learning after broad lesions to lateral prefrontal cortex. However, rather than decreasing the negative GRS effect as in humans, lateral lesions *increased* the positive GRS effect in macaques (Fig.6). One interpretation could argue that the GRS effect, together with the decline of local reward learning, reflects a task-adaptive process in humans, that matures during adolescences and is compromised by lesions. By contrast, in monkeys, the GRS effect could reflect a suboptimal ‘spread’ of reward which is even increased by lateral frontal lobe lesions. However, an alternative account could argue that the overall positive shift in GRS influence on choice after lesions in both humans and monkeys reflects a general role for the lateral frontal cortex in contextualizing the GRS to avoid or suppress the influence of spread of effect mechanisms. In humans, this suppression is strong enough to produce a negative GRS effect, but it is less influential in macaque choices.

2. The authors might also consider adding a **citation diversity statement** (see Zurn, Bassett, & Rust 2020 Trends in Cog. Sci. for rationale, as well as references to original work and a template and tools for implementation) to help combat systemic inequalities in academia.

Thank you for this suggestion. We have now included a citation diversity statement as follows:

*Recent work has identified a bias in citation practices which results in papers from women and other minorities being under-cited. The citation diversity metrics below, proposed by Zurn and colleagues [102] encourage us to proactively reflect on our citation practices and we have taken steps to ensure a more accurate reflection of the diversity in science. By this measure (and excluding self-citations to the first and last authors of our current paper), our references contain 19.8% woman(first)/woman(last), 14.6% man/woman, 17.2% woman/man, and 48.3% man/man.*

**Minor grammatical changes:**

1. In general, there were more typos and grammatical errors in this version of the manuscript (as often happens in revisions). I would recommend a thorough pass on the language, but here are a few I happened to write down while reading:

   Abstract:
   *       "anterior insular cortex" when insula is used as an adjective
   Introduction:
   *       Missing word in "more rapid maturation of subcortical ___"
   *       Generally watch for commas needed between sentences, e.g., after refs [35,36]

   Results:
   *       "The second reason was to examine, for the first time, whether changes in global reward learning were also apparent after lateral frontal lobe LESIONS in macaques, like in our human lesion data.
   Discussion:
   *       "(Fig.4,5). This suggests that …"
   *       ", in increased the effect in macaques."

   Supplement:
   *       S4 caption: "Consequently, in the analysis, variance associated with one learning mechanism is "

*All of the above have been resolved and we have proof-read the carefully for others.*
